# Materiality, Agency and Evolution of Lithic Technology: an Integrated Perspective for Palaeolithic Archaeology

**DOI:** 10.1007/s10816-020-09483-6

**Published:** 2020-09-03

**Authors:** Shumon T. Hussain, Manuel Will

**Affiliations:** 1 Department of Archaeology and Heritage Studies, Aarhus University, Moesgård Allé 20, 8270 Højbjerg, Aarhus, Denmark; 2 CRC 806 ‘Our Way to Europe’, University of Cologne, Cologne, Germany; 3 Centre for Environmental Humanities (CEH), Aarhus University, Aarhus, Denmark; 4 BIOCHANGE – for Biodiversity Dynamics in a Changing World, Aarhus University, Aarhus, Denmark; 5 Centre for Interdisciplinary Research (ZiF), University of Bielefeld, Bielefeld, Germany; 6 Department of Early Prehistory and Quaternary Ecology, University of Tübingen, Burgsteige 11, 72070 Tübingen, Germany

**Keywords:** Human evolution, Stone artefacts, Non-human turn, Material agency, Object-scapes, Transdisciplinarity, Deep past

## Abstract

Considerations of materiality and object-oriented approaches have greatly influenced the development of archaeological theory in recent years. Yet, Palaeolithic archaeology has been slow in incorporating this emerging body of scholarship and exploring its bearing on the human deep past. This paper probes into the potential of materiality theory to clarify the material dynamics of the Plio-Pleistocene and seeks to re-articulate the debate on the evolution of our species with materiality discourses in archaeology and the humanities more broadly. We argue that the signature temporalities and geospatial scales of observation provided by the Palaeolithic record offer unique opportunities to examine the active role of material things, objects, artefacts and technologies in the emergence, stabilisation and transformation of hominin lifeworlds and the accretion of long-term trajectories of material culture change. We map three axes of human–thing relations—ecological, technical and evolutionary—and deploy a range of case studies from the literature to show that a critical re-assessment of material agency not only discloses novel insights and questions, but can also refine what we already know about the human deep past. Our exploration underscores the benefits of de-centring human behaviour and intentionality and demonstrates that materiality lends itself as a productive nexus of exchange and mutual inspiration for diverging schools and research interests in Palaeolithic archaeology. An integrated object-oriented perspective calls attention to the human condition as a product of millennial-scale human–thing co-adaptation, in the course of which hominins, artefacts and technologies continuously influenced and co-created each other.

## Introduction

Palaeolithic archaeology is conventionally defined as the study of the behaviour and lifeways of hominins during the earliest periods of prehistory, addressing the enormous range of preliterary and presedentary human experience from the emergence of the first stone tools between 3 and 4 million years ago to the end of the last Ice Age about 11,800 years ago. As perhaps no other field of prehistoric investigation, Palaeolithic research relies strongly, and often exclusively, on the patchy record of stones surviving the thousands or even millions of years of combined human and geological history. Despite the apparent over-abundance of material evidence—artefacts and ecofacts—relative to hominin fossils and biosignatures, Palaeolithic archaeologists commonly conceive of their research enterprise as the examination of hominin behaviour or the study of hominin–environment interactions. This approach places the focus of inquiry either on hominins or on their attendant ecologies and natural environments. The surprising result is a long-standing difficulty to define and negotiate the role and place of material things in the evolution of our species.

The ongoing discourse on lithic technology illustrates this dilemma: Stone tools are either taken to be intimately tied up with hominin biology, behaviour and culture—*representing* or *reflecting* hominin activity, sociality and cognition—or are regarded as a potent adaptive interface, alleviating the tension *between* hominins and their palaeoenvironments. The result is an obvious paradox: Even though the pre-Holocene record is made up almost exclusively of material remains, Palaeolithic archaeologists seem to have largely neglected the evidential value of things and objects themselves and have hitherto invested only little into the development of conceptual and methodological resources for exploring the foundational materiality of their record. This paper attempts to fill this void and takes some initial steps to explore the significance of material things, objects and artefacts through the lens of materiality theory and object-centred epistemologies. We argue that the consideration of material agency opens up new research avenues and subverts long-perpetuated narratives in the field, re-calibrating our recognition and understanding of the active role of materials and things in human life, culture and evolution.

We begin with an overview of key insights and concepts gathered under the umbrella of materiality theory which can be co-opted for the study of the human deep past. Subsequently, we re-visit the nature of the Palaeolithic record and discuss the advantages of employing materiality-based approaches, followed by a selective review of ongoing research into the Late Pleistocene of Western Eurasia illustrating the benefits of object-oriented perspectives on macro-archaeological patterns and the formative role of material environments. We then turn to lithic technology as the most abundant line of Palaeolithic evidence and identify three complementary axes of inquiry which profit from materiality considerations. Drawing on examples from Europe, Asia and Africa, we showcase how a focused concern with the agency, impact and consequences of material things and technologies can inform new approaches and interpretations in disparate domains of research, such as ecological landscape archaeology, lithic reduction systems and the study of the millennial-scale evolution of stone artefacts. We finally review our integrated perspective and assess the promises of debating materiality in the wider field of human origins studies. We conclude with a plea for paying more attention to the many voices of material things and underscore the ability of object-centred approaches to bridge divergent disciplinary perspectives, acting as a nexus of concerted research into the material conditions of life, culture and evolution in the non-analogous Palaeolithic past.

## Materiality: a Brief Recap and Overview of Key Concepts

Materiality conveys a fundamental re-conceptualisation of material things, objects, artefacts and technologies, which are no longer regarded to merely *represent* or *reflect* human behaviours and thoughts or to *articulate* ecological needs, but instead come into view as constituents, stimuli and catalysts of human life and culture themselves (Henare *et al.* 2007; Carlile and Langley 2013; Lemonnier 2014). Materiality draws attention to the ‘thingness’ of things, the ‘objecthood’ of objects and the ‘artefactuality’ of artefacts, flagging up the possibility of material factors to make a real difference for people’s lives, behaviours and thoughts (Meskell 2005; Jones and Boivin 2018). Broadly conceived, materiality theory seeks to overcome ‘hylomorphism’ (*cf.* Johnston 2006)—the ancient Greek dualism between ‘form’ (*eidos* or *morphê*) and ‘matter’ (*hulê*)—and the influential Descartian rendering of things as *res extensa*. Instead, material things are recognised as actively contributing to the makeup of shared lifeworlds and as vital media of thought and action, as much ‘constituting’ as ‘constituted’ (Tilley 2007). Materiality theory acknowledges the agency of things and larger object assemblages and probes into their power to shape human realities and calibrate long-term trajectories of biocultural evolution (Knappett 2005; Boivin 2008; Soentgen 2014).

Materiality thinking is motivated by a number of recent theoretical re-orientations within the humanities and social sciences, most notably the ‘material’ and ‘non-human’ turns (*cf.* Miller 1998; Grusin 2015) founded in a resolute critique of long-standing pre-occupations with human exceptionality and human-centred explanations. Object-oriented approaches are deployed to de-centre the human (Hayles 1999; Knappett and Malafouris 2008; Bogost 2012) and to examine the consequences of changing and internally differentiated material worlds for human behaviour, culture and evolution (Bennett and Joyce 2010; Hicks 2018). The renewed surge of scholarly interest in the *material conditions* of human life is additionally fuelled by recent developments in *material culture studies* (*e.g.* Miller 1998, 2005; Hahn 2005, 2015; Tilley *et al.* 2006a; Eggert 2014, p. 26; Hicks and Beaudry 2018) or *science and technology studies* (STS; *e.g.* Kirchhoff 2009; Pickering 2010), and predicated upon wider intellectual currents and movements in the humanities such as *new materialism* (*e.g.* Coole and Frost 2010; Bennett 2010; Dolphijn and van der Tuin 2012), *speculative realism* (*e.g.* Meillassoux 2008; Bryant *et al.* 2011; DeLanda 2016) or what is referred to as *post-humanism* (*e.g.* Latour 1991; Descola 2005; Haraway 2007, 2016; Braidotti 2013; Ferrando 2013). These movements seek to defuse entrenched subject–object dichotomies and to re-visit taken-for-granted power relations and chains of determination in order to enable a more complete understanding of the many complexities of human–thing relations. By setting aside passive and inert conceptualisations of matter, such perspectives embrace the continuous co-constitution of humans and things (Tilley *et al.* 2006b, p. 2; Orlikowski 2007; Barrett 2014): Material things, in this view, create people as much as people create things (Tilley 1999; Gosden 2005, 2006; Robb 2007; Warnier 2009; Hussain 2018b).

In archaeology, aspects of materiality theory inform at least four major strands of contemporary scholarship: (1) Object-centred and ‘symmetrical’ archaeologies (*e.g.* Olsen 2003, 2010, 2012; Gosden 2005; Olsten *et al.* 2012; Shanks 2007; Webmoor 2012; Nativ 2018 and subsequent discussion); (2) archaeologies of ‘entanglement’ (*e.g.* Hodder 2011, 2012, 2014; Der and Fernandini 2016) and their ‘relational’ confederatives (*e.g.* Robb 2013; Watts 2013; Buchanan and Skousen 2015); (3) ‘more-than-human’ or ‘multispecies’ archaeologies (*e.g.* Pilaar Birch 2018); and (4) a range of cognitive approaches based on material engagement theory (MET) and cognate perspectives (*cf.* Renfrew and Malafouris 2010; Wheeler 2010; Malafouris 2013, 2015, 2016). These approaches are united by the basic recognition that material things represent *nodal points* in the complex web of past realities (Knappett 2014). With the notable exception of MET, however, these object-centred perspectives have so far developed an impact only on post-Pleistocene archaeologies and remain largely marginalised within human origins research (but see Chazan 2018 for an exception).

In what follows, we explore the anchoring notion of material agency and discuss some ideas and concepts which help to clarify the active involvement of material things in human affairs. These notions supply a basic terminology for analysing the potency of objects in the past and ground the subsequent exploration of material significances in the Palaeolithic.

### Material Agency

The activity and consequentiality of material things is often addressed under the rubric of ‘material agency’, and various perspectives on the nature and role of the latter have been developed in recent years within archaeology and other object-invested academic disciplines (*e.g.* Henare *et al.* 2007; Knappett and Malafouris 2008; Kirchhoff 2009; Pickering 2010; Lindstrøm 2015; Jones and Boivin 2018). This diversity of approaches provides the required flexibility, nuance and specificity to explore similarities and differences in how things interact with humans and variously shape human culture, cosmology and sociality. The rooting idea of material agency should therefore be discussed against its broader multi-national and interdisciplinary background, engaging with the various research traditions and paradigms that have made an effort to disentangle the manifold material dimensions of human societies past and present. *Material culture studies* and French techno-anthropology have demonstrated that any attempt to understand, map and compare material agencies depends on a careful consideration of the various aspects and dimensions of thingness and objecthood, distinguishing between ‘objects’, ‘things’, ‘artefacts’, ‘instruments’, ‘tools’ and ‘technologies’ among others (*e.g.* Rabardel 1995; Hahn *et al.* 2014, p. 1–3; Guchet 2018). As Guchet (2018, p. 238) notes, ‘[w]hile *artifacts* are above all man-made, intentional entities’, technological *objects* are ‘beings-in-the-world among other entities: beyond the intentions of their makers, technological objects prove to have an activity of their own in the natural and the social world. They link to other objects – be they natural or artifactual – in an unpredicted way, and may give rise to processes that escape our control.’

Hahn (2005, p. 18; translation of the authors) similarly cautions against a narrowing of analysis in favour of the intentional products of human behaviour and technical action and proposes to examine the ‘totality of material objects’ that ‘play a role in the human lifeworld’. Symptomatically, Hahn (2005) stresses the context dependency and fuzziness of material significance, giving way to the inherent *polyvalency* of material objects, transforming the human experience and drawing humans into ever-changing material engagements. Importantly, this material polyvalence differs from semantic incompleteness or intertextuality as encountered in human languages, undermining language as an appropriate metaphor for the study of human–thing relations. Object-centred approaches thus *counter* the long-standing notion of material culture as text and seek to investigate the material world as a self-contained field of reality, possibly endowed with its own logic and rule sets. The implication is that materiality theory works towards an understanding of the difference-making propensity of material things that goes beyond the idea of the medium.

#### Affectivity and Material Possibility

Shifting the attention from humans to material objects inevitably leads to a critical re-examination of taken-for-granted notions of causality and agency. Without denying the causal role of human behaviour in the construction, modification and perpetuation of material configurations, materiality theory undercuts the idea that objects are lifeless and passive. Instead, it promotes a distributed or network-theoretical model of production and formation in which multiple heterogeneous entities continuously interfere with one another and bring their specific properties, possibilities and constraints into play to make a difference in the world. This concept of agency—compatible with the proposal of actor–network theory (ANT; *e.g.* Latour 2005)—is less demanding than the traditional humanistic account of action, *e.g.* transpired by the German term *Handlung*, and does not necessary rely on thick concepts of intentionality. This important re-conceptualisation of agency—foregrounding the *difference-making capacity* of agents (*cf.* Pearson 2013, p. 134)—overcomes the object anarchism that inheres in most anthropocentric models of action, acknowledges the complexity of real-world pattern formation and emphasises the multiplicity and context dependency of field-specific involvements in worldly affairs. Latour’s (1991, 1994, 2005) notion of the ‘actant’ is consistent with this understanding, recognising the specificity of human agency without overlooking the many other reality-making beings-in-the-world, including material objects. Two distinctions help to illustrate this expansion and re-configuration of agency: *effect* vs. *affect* and *direct action* vs. *action at a distance*.

While material objects often become causally effective only through human initialisation, activation and instrumentalisation, their physical makeup and relationship to other things has the propensity to *affect* those who use, engage or live with them (Hamilakis and Jones 2017). Affect is understood here as a ‘relay between subject and object’ (Houser 2018, p. 16): Objects can invite, afford and structure human actions and disrupt or inhibit others (*e.g.* Gibson 1979; Reed 1988, 1996; Lemonnier 2014; Keane 2018), stipulate metaphors and cognitive associations (Coward and Gamble 2010) and thus influence the contents and modes of thought (Boivin 2008; Wynn and Coolidge 2017; Malafouris 2020), but also draw people into direction-giving webs of interdependency (Hodder 2012). Gell’s (1992, 1996, 1998) influential analysis of agency accentuates this affective capacity of non-human things, notably their ability to ‘enchant’ and ‘entrap’ people in human–thing dialectics. The affectivity of material things—in contrast to strict causal efficacy—indirectly and often subtly regulates how humans *attend to* things and how pre-furnished thing-worlds alter or pre-structure the behavioural space of their human counterparts. Object agency is thus often a matter of *action at a distance*, rather than direct action, and has less to do with achieving a predictable effect than opening up a space of potentiality for optimisation, modification, co-optation and change.

#### Vibrancy and Conactivity

Although power relations between humans and things are rarely symmetrical, the agency of things and artefacts remains irreducible to their human delegates. In Bennett’s (2018) terms, objects possess ‘material vibrancy’ that escapes human surveillance and control, yet modulates and directs human–object engagements. For Bennett (2010), agency denotes a *relational* concept, rather than something to be located in a single bounded, spatiotemporal entity. We may thus speak of ‘confederate agency’ (Bennett 2010, p. 23) or simply an activity that derives from the interconnection of a plurality of difference-making entities. Together, these heterogeneous entities, involving both humans and material things, enact a distributed but collective form of agency—what Bennett (2010), who specifically discusses minerals and rocks at some length, baptises *conactivity.*

Conactivity underscores the primacy of human–thing connections and the reciprocity implied in human interactions with the material world. Bennett (2010, p. 1, 2004) distinguishes between *positive* and *negative* thing-powers. Negative thing-powers comprise the constraining and limiting effects of things: They introduce resistance to human life and narrow down the horizon of what appears *possible* and *feasible*. This negative agency of things is grounded in the fact that people are always inescapably born into repleted material worlds, which in turn mould human perceptual, cognitive and behavioural fields as well as the likelihood and nature of material discovery, innovation and modification. Positive thing-powers, by contrast, convoke the ability of materials to ‘make things happen, to produce effects’, transcending the mere affectivity of non-human objecthood (Bennett 2010, p. 5). These material effects depend on the unique ways-of-being linked to specific non-human objects—their affordances, inherent tendencies and trajectories of change and decay—and the myriad ways in which the target objects continuously forge and renew relationships with human and non-human entities and thereby steer stabilisation or re-organisation within shared lifeworlds. The agency of things, in this view, is an *ecological* force regulating how humans intersect with their environments, and helps to understand how novelty grows out of ever-changing human–thing articulations—material things are, as Ingold (2012) reminds us, deeply ‘enrolled in human form-making processes’.

The conactive model of agency conveys a critique on the notion of the *artefact*, so routinely invoked by archaeologists to map and explain the human past (*e.g.* Wynn 1995; Pelegrin 2005; Pelegrin and Roche 2017) and so readily subverted by those who rank impersonal, especially biological and environmental forces, higher in the ladder of causality and explanation (*e.g.* Rolland and Dibble 1990; Corbey *et al.* 2016). Within a conactive world, however, there is little space for artefacts proper—forms that *exclusively* derive from the imposition of human will into mute matter (*cf.* Ingold 2013, p. 37)—and artefacts always result from the mutual infusion, orchestration and interlocking of human and non-human factors: Humans and artefacts ‘interimplicate’ each other (*sensu* Butler 2004). This point is of crucial importance for it offers a new inroad to the problem of intentionality and technical concept formation in earliest human prehistory: It is neither reliant on the conceptually flawed polarisation of form and matter (hylomorphism), nor on pitching the human against the non-human, as *e.g.* reflected in the ‘finished artefact fallacy’ (Davidson and Noble 1993; Dibble *et al.* 2017). Negating material resistances always leads to the disintegration of the respective materials, and stone workers must thus—to paraphrase Deleuze and Guattari (2004, p. 450–451)—at least in part ‘surrender’ to the physical qualities of the worked materials and ‘follow where [they] lead’.

#### Assemblages and Intra-action(s)

Another gateway into examining the varying effects of material configurations on human life, behaviour and evolution is opened up by assemblage theory (AT). AT is rapidly gathering a significant following in the humanities and social sciences and has generated new ways of unpacking the complex interlocking of socio-material realities (*e.g.* Marcus and Saka 2006; DeLanda 2006, 2016; Webb 2009; Allen 2011; Butler 2015; Hamilakis and Jones 2017; Jervis 2018). There are at least three major conceptual inputs that delineate this broader field: (1) Freud’s (1985) idea of the ‘complex’; (2) Deleuze and Guattari’s (1983) notion of the ‘assemblage’; and (3) Foucault’s (2005) concepts of ‘power’ and ‘governmentality’ (*cf.* Buchanan 2015). In general terms, AT orientates material inquiry towards the diversified, heterogeneous and conflict-ridden character of reality—a reality in which multiple, potentially equipotent but often quite disparate entities, mesh, interact, resonate, ignore, plunge and/or overthrow each other (*cf.* DeLanda 2016). Assemblages—etymologically derived from the French word *agencement* (Phillips 2006)—refer to multi-dimensional articulations of things, ideas and forces, which dispose of ‘operational totality’ (*cf.* Law 2004, p. 41). These configurations of deviant potencies always ‘work’ as a unit, yet lack an all-absorbing coherence so that the internal structure of the total configuration cannot dissolve entirely. The resulting texture of these totalities, in the terminology of Deleuze and Guattari (1980, p. 9–37), can thus be described as ‘rhizomatic’.

AT introduces a precipitous and often catastrophic dynamism to processes of social evolution: Social realities are regarded as *imperfect* orchestrations of distinct tendencies and difference-making voices, and varying domains and sub-domains of reality come into view as suspenseful assemblages and sub-assemblages. The implied *infrastructural view* of reality—involving nested hierarchies and precarious layerings—counters an intuitive understanding of assemblages as mere aggregates or fitting material compositions (*contra e.g.* Dibble *et al.* 2017; Rezek *et al.* 2020 in the context of lithic studies). AT puts emphasis on the ability of individual assemblage parts to play out their material predispositions and draw other parts into their field of influence (*cf.* DeLanda 2015). The internal friction that is in this manner continuously created, re-directed and translated delivers a recurrent impetus for change and transformation. From the perspective of assemblage thinking, alteration and metamorphosis constitute the *modus operandi* of reality, while stability and stasis are exceptional conditions in need of explanation. For the same reason, the perpetual negotiation and re-arrangement of *power relations* among parts emerges as a key vector of assemblage formation, diffraction and reproduction.

AT offers a toolbox for re-describing the interplay between the emergence of material structures and the agency of the involved parts. Thing-powers can be monitored through the aptitude of objects, artefacts and technologies to re-configure the web of relationships giving shape to the larger material infrastructures in which they participate: The latter’s ongoing, dynamic renewal and modification is mediated by the ‘self-organisational’ tendencies of heterogeneous assemblages (DeLanda 2000, p. 16, 2016), but also by the materiality of its constituent objects which variously ‘enthral’ or even ‘enslave’ each other, initiate uni- or bidirectional dependencies, supress or re-direct the developmental trajectories of other entities and enter positive co-evolutionary alliances. These shifting and evolving possibilities of object-articulation in a given assemblage depend primarily on the material qualities of the attendant things, objects, artefacts and technologies: They specify what can *emerge* at the intersection of heterogeneous parts. AT focusses on both the difficulties and possibilities of *material coordination* in a messy and complicated world and makes a strong case for the reciprocal dependency of structured wholes and the materiality of counteracting parts. From an evolutionary perspective, AT fosters the examination of interlinkages between the ‘being’ and ‘becoming’ of shifting material configurations and provides fresh resources for understanding cyclic processes of assemblage differentiation, re-organisation, mutation and integration. AT foregrounds the study of ‘intra-action’ (*sensu* Barad 2007, p. 141) as a counter-concept to ‘interaction’—assemblage–internal forces cross-calibrating, diffracting and influencing each other to bring about ever-changing and differentiated yet inseparable wholes.

Moreover, there is an as of yet unrecognised possibility of bringing AT into productive dialogue with emerging perspectives from community ecology, such as those subscribing to community assembly theory (Weiher *et al.* 2011) or co-existence theory (HilleRisLambers *et al.* 2012). These viewpoints also take complex assemblages composed of diverse ecological actors as their point of departure and deploy specific quantitative methods to examine interactive dynamics within and between respective assemblages, such as filtering, clustering, niche partitioning, differentiation and stabilisation, as well as neutral vs. niche-based sorting. Analogous to the variants of AT in the humanities and social sciences, community ecology approaches rest on the idea of ecological friction and the resulting need for coordination and foreground multi-dimensional structural dynamics in the formation and reproduction of ecological realities. Some of these concepts and perspectives may hence be co-opted to enrich and refine our understanding of shifting ‘object-regimes’ (Baudrillard 1968; Thévenot 2001) or mutating ‘object ecologies’ (Bennett 2010; Hörl 2017) in human evolution.

## A Call to Arms: the Nature of the Palaeolithic Record

The Palaeolithic harbours material evidence for over 3 million years of human evolution in which preliterate populations inhabited diverse Plio-Pleistocene climates and landscapes and developed the biosocial preconditions for modern human life and culture (Shryock *et al.* 2011). In contrast to fields such as later prehistory, history or socio-cultural anthropology engaged with ‘shallow-time’ (*cf.* Happel 1996), Palaeolithic archaeology is a ‘deep-time’ discipline whose subject matters and targets of analysis are far removed in time and whose observational focus tends to collapse many thousands of years (Hussain and Riede 2020). Much of the available Palaeolithic evidence is a product of survivorship bias (*cf.* Budd and Mann 2018) as well as negative filtering and information-destroying processes operating on varying and often vast temporal and spatial scales. It is widely accepted—and shown empirically (Surovell *et al.* 2009; Locht *et al.* 2016)—that both the *quality* and the *resolution* of the Palaeolithic record differ markedly from younger periods (Meltzer 2004; Perreault 2019, p. 132–133). The Palaeolithic archaeological record is a co-production of a uniquely broad range of compounding factors and processes which either re-configure material traces—*e.g.* through various kinds of mixing (Perreault 2019: Chapter 3) and time-averaging (Stern 1993; Bailey and Galanidou 2009)—or prevent such traces from entering or residing in the record (Gamble 2001, p. 67–72; Hussain 2018a, 8–13). In addition to these geogenic or taphonomic processes, cultural and behavioural filtering can also play a generative role in the formation of the record (Schiffer 1976; Bernbeck 1997, 67–81).

Yet, the distinct material architecture of the Palaeolithic is not only a consequence of negative interference, impoverishment and overprinting, but also emerges from human ecological, behavioural and cultural dynamics that can be very different from anything recorded in later periods. Notwithstanding, the Palaeolithic archaeological record joins its palaeontological counterpart in what Currie (2019, p. 63) has recently described as deep-time ‘pecularity’. This term encapsulates the processual opening, modification and closure of various possibilities, potentialities and constraints of development which account for the observable Palaeolithic patterns, insofar as ‘previous conditions laid the groundwork for, enabled, dampened and triggered later conditions’. Doing justice to this foundational peculiarity of Palaeolithic archaeology as the discipline of the human deep past—acknowledging materiality, contingency and historicity—requires not only to collapse the boundaries between ‘nomothetic’ and ‘idiographic’ science, but also to strengthen the examination of the basal *conditions of material possibility* (Currie 2019, p. 62–63). Materiality perspectives help to enrich and deepen our understanding of this possibility space and to trace some of its otherwise difficult-to-grasp outlines.

Even though the long-term macro-archaeological perspective afforded by the Palaeolithic archaeological record is recognised to promote unique insights by some scholars (*e.g.* Boëda 2005; Gamble 2015; Perreault 2019, p. 170–172), the large majority of researchers in the field perceives the nature and structure of the record with its many gaps and uncertainties as a serious obstacle to in-depth knowledge about the deep past (*cf.* Gamble 2001, p. 67–72; Boissinot 2011). In relation to more recent archaeological periods such as the Neolithic or Bronze Age, the Palaeolithic record is regularly re-cast as a curtailed, skewed and fragmented, hindering or at best considerably complicating access to the specificities of past human life and culture. Symptomatically, Hodder (2011, p. 164) has noted *en passant* the comparatively low degree of object–object and human–object entanglements characterising what precedes the Holocene. This implied inferior condition of the record has certainly influenced the prevailing perspective of a reduced utility of materiality approaches for the study of the Palaeolithic period. Despite occasionally outstanding Pleistocene conditions of preservation and sites offering high-resolution snapshots of past hominin daily routines such as FLK 22 (Blumenschine *et al*. 2012), Abri Romaní (Carbonell 2012), Pincevent (Julien and Karlin 2014) or Monruz and Champréveyres (Cattin 2010), the dominant and arguably problematic view is still that Palaeoltihic signatures are inherently deficient and hardly competitive with those left behind by more recent periods.

This ostensible knowledge-restraining quality of the Palaeolithic record is regarded to undermine the productive application of concepts and perspectives drawn from putatively demanding bodies of theory from the humanities and cultural sciences such as materiality (*cf.* esp. Davies 2000). When getting to grips with the character of the pre-Holocene record, this stigmatisation of interpretive excess ranks among the chief reasons why materiality and material agency are so rarely discussed in relation to the human deep past. While materiality theory and object-centred perspectives have now made their way into the mainstay of archaeological theory—as showcased by the many edited volumes, textbooks, encyclopaedia entries and introductory compendia featuring the topic (DeMarrais *et al.* 2004; Tilley *et al.* 2006a; Hodder 2012; Olsen 2010; Knappett 2012, 2014; Olsen *et al.* 2012; Robb and Harris 2013; Witmore 2014, 2017)—issues of material agency and human–thing relations have at best been delegated to the periphery of the discourse on human origins and the Palaeolithic past.

The development of object-centred approaches to the Palaeolithic is further complicated by divergent pre-occupations and concerns perpetuated by the dominant research trajectories in the field. Anglophone Palaeolithic archaeology has for example cultivated a set of data-driven and strictly ‘analytic’ perspectives (*sensu* Hussain 2018b; *cf.* Binford and Binford 1968; Clarke 1968; Shennan 2004; Lycett and Chauhan 2010) and is often hostile against concepts and theories with a perceived affinity to ‘post-processualism’ or interpretative archaeologies writ large (*cf.* Davies 2000; Shea 2011). The Anglophone tradition also tends to align with goals of research programmes such as *human origins*, *palaeoanthropology* or *evolutionary archaeology*, which variously champion ecological or Neo-Darwinian evolutionary research frameworks to investigate the Palaeolithic past (Kuhn 2004; Shennan 2008; Shea 2011; Goodale and Andrefsky 2015). For many Anglophone practitioners, there is neither the need nor the space for another principal corpus of theory to be incorporated, discussed and explored, especially since the terminology and founding assumptions of materiality thinking seemingly conflict with received wisdom in these circles. What is easily overlooked, however, are some notable conceptual convergences between materiality theory and evolutionary approaches in Palaeolithic archaeology, especially those sympathetic with the ambitions of the ‘extended evolutionary synthesis’ (EES; see below for an explicit discussion).

Palaeolithic scholars working in the French tradition broadly conceived—especially those who identify themselves with the transdisciplinary enterprise of *Technologie* (Leroi-Gourhan 1949; Perlès 1974; Tixier 1980; Haudricourt 1987; Inizan *et al.* 1995, p. 13)—are more sympathetic to the core ideas advocated by the ‘material turn’ and often make implicit use of cognate concepts (*e.g.* Pesesse 2018; *cf.* esp. Warnier 1999; Sigaut 2012; Lemonnier 2014 for some of the relevant background literature). At the same time, and ironically partly because of this, however, these researchers tend to have difficulties with recognising the conceptual novelty of materiality thinking, also because the respective body of theory is easily regarded as yet another instance of ‘high-level’ theorising which French technologists are generally suspicious about (*cf.* Audouze 1999; Hussain 2018a, p. 121–124).

Despite of these reservations, we argue that the very nature of the pre-Holocene archaeological record lends itself to materiality considerations and object-centred research: A large portion of the Palaeolithic record de facto *consists of* material things, such as artefacts, ecofacts and sediments, and the record is virtually a *deep-time document of changing thing and object configurations* (*e.g.* Conneller 2011). Hominins and their behaviours are almost never directly observable and their actions and cognition must always be inferred or approximated from material traces or biosignatures. Albeit perhaps trivial at first glance, this point cannot be overemphasised since hominins are still viewed as the unrivalled protagonists and observables of the past, while things and objects are seen to represent merely residuals, expressions or even epiphenomena of their actions, beliefs and cultural deeds.

What is perceived as a deficiency of the Palaeolithic record may thus be recognised as an enabling condition of knowing the materiality of the deep past. The various filtering, information-destroying and mixing processes co-creating and curating the material record of the Palaeolithic introduce dynamics and effects that escape the grasp and control of human agency, anticipation and will. The Palaeolithic record thus furnishes a compelling corpus of evidence for studying the contingent influence of things and material worlds on human behaviour, cognition and evolution over many thousands of years. Since the impact of human actions and thing-powers expresses itself on divergent temporal scales (*e.g.* Simondon 1958; Hodder 2014; Guchet 2014) and since the pre-Holocene record naturally downplays, and at times even obscures, short-term changes and individual-level behavioural signatures (Stern 1993; Holdaway and Wandsnider 2008; Bailey and Galanidou 2009), the Palaeolithic record can be re-cast as a powerful means to re-direct the attention to the framing capacity and proactive role of objects in early prehistory and to chart their long-term contribution to the human story. With Palaeolithic eyes, we may begin to trace and interrogate the propensity of things, objects and artefacts to make and contribute to human history (*cf.* Boëda 2005; Bailey 2007; Vaquero 2008) and how their field-specific agency, historicity and ‘peculiarity’ (*sensu* Currie 2019) relates to, collides or interferes with the history-making efforts of their human sparring partners.

A final reason why materiality thinking promises to open up fruitful research avenues in Palaeolithic studies is the comparative, multispecies nexus provided by archaeological investigations of the deep past: The material record of the Palaeolithic is the only entry point to a past where hominin biocultural diversity was a decisive factor. It features multiple hominin species or demes of which several *Homo* and possibly *Australopithecus* groups regularly produced and utilised stone tools (Harmand *et al.* 2015; Shea 2017; Herries *et al.* 2020). Archaeological and palaeoanthropological research has shown that this heightened hominin diversity was the *status quo* in human evolution until at least 100,000 years ago and likely until much later (*e.g.* Wood and Boyle 2016; Foley 2018; Rizal *et al.* 2020). Studying the Palaeolithic therefore enables an inter-species approach to the intersectionality of hominin bodies, minds and material cultures on evolutionary timescales (*cf.* Coward and Gamble 2008). Materiality theory thus not only supplies promising resources to examine the co-constitution of objects and hominins, Palaeolithic research has also the chance here to make a substantial contribution to the wider ‘material turn’ in the humanities and social sciences by introducing a multispecies perspective in human–thing studies and to begin to empirically map the potentialities and ramifications of fossil human–thing articulations and the role of the material in the making of different humanities.

**Fig. 1:**
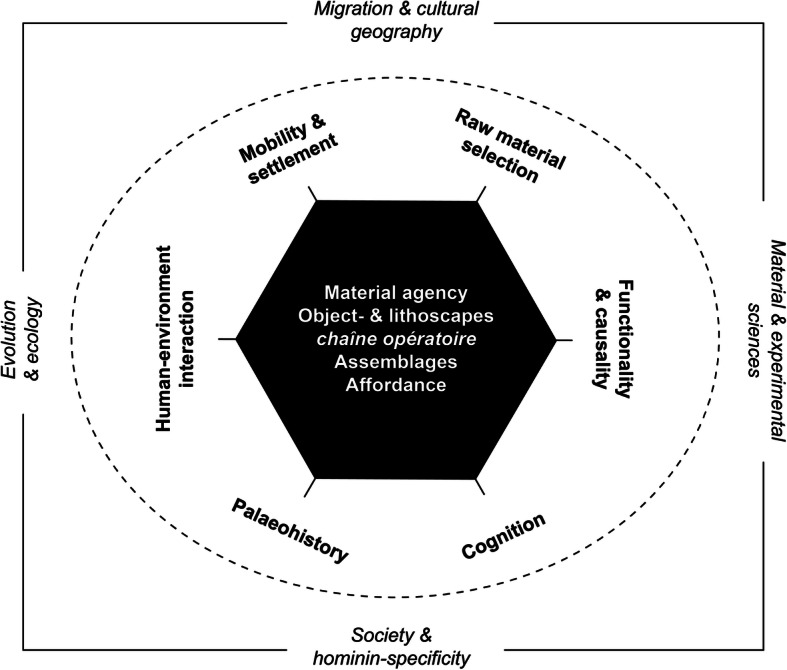
The nexus of materiality in relation to selected thematic concerns in Palaeolithic archaeology. The interior hexagon gathers key dimensions/opportunities of materiality-based inquiry, while the outer perimeters exhibit other domains of Palaeolithic research which can (more indirectly) benefit from object-oriented perspectives. Distance from the centre signals a gradual loss of interpretative significance. The figure offers a schematic and provisional roadmap of how materiality theory may be articulated with traditional domains of Palaeolithic investigation

Materiality thinking and object-centred approaches re-invigorate long-standing debates in Palaeolithic archaeology inspiring new questions and dismantling old narratives, and also offer a flexible toolbox of concepts and critical perspectives from which different research projects may equally draw (Fig. 1). While varying research trajectories in the field are likely to regard different aspects of the toolbox useful, they may nonetheless agree on shared problems and overarching research concerns with regard to the material past. A broad and inclusive understanding of materiality rooted in epistemological pluralism provides a common discursive platform, allows individual practitioners with varying backgrounds to explore relevant issues at the human–thing interface and to take advantage of the merits of their differential viewpoints. Shielding the Palaeolithic against materiality theory cripples our ability to develop a multi-faceted understanding of the human deep past and threatens to create a sense of this past as somewhat ‘de-materialised’ human experience. The danger is thus to return to a linear narrative of human evolution in which materiality is treated as a somewhat late graft, added only later to the essential human which evolved in the thickets of ‘deep time’ (*sensu* Shryock *et al.* 2011).

## ‘Object-scapes’ and Long-Term Assemblage Dynamics in Late Pleistocene Western Eurasia

The ‘human niche’ (Stout 2011; Gamble *et al.* 2014; Haidle *et al.* 2015; Shea 2016, 2017) is reflected in diagnostic material environments—for White (1949), Binford (1962, p. 218) and others both the source and locus of human ‘extra-somatic’ adaptation. At least since hominins began to systematically manufacture sharp-edged stone artefacts between ca. 3 and 4 million years ago (Harmand *et al.* 2015; Braun *et al.* 2019), human life is bound to diversified and structured worlds of material things, which bring in their own logic, affordances and possibilities and alter long-term evolutionary dynamics (*cf.* Stiegler 1994; Thévenot 2001; Lemonnier 2014; Hussain 2018b). Being human is the result of millennial-scale hominin–thing interactions, which have irrevocably transformed both hominins and things (Leroi-Gourhan 1943/1945, 1953, 1964/1965; Barrett 2014). The evolving confederacy of hominins and material things, objects, technologies and artefacts thus emerges as a key factor in human evolution. Hominins must not only be expected to have developed ‘imagined communities’ (*sensu* Anderson 1991) with their conspecifics or animal co-dwellers (*cf.* Hussain 2019), the communities they have addressed, experienced, cultivated and imagined were also object-enriched. Although some non-human animals also utilise or produce objects, the hominin lineage remains unique in the scope, diversity and complexity of supported material culture relations (Laland *et al.* 2008; Sigaut 2012).

This ‘more-than-human’ understanding of the human deep past motivates the longitudinal and cross-sectional investigation of a general ‘ecology of things’ (Hörl 2017), fuelling and orienting long-term developments in both hominin and object domains. Changes in hominin *object ecology* can have enormous ramifications for the pattern, directionality, mode and tempo of human biocultural evolution (*cf.* Boivin 2008; Tomlinson 2018; Chazan 2018). In analogy to the role of physical and animate landscapes in human evolution, the respective collations and assemblages of difference-making material things may be addressed as ‘object-scapes’ (Versluys 2017). Object-scapes are ‘repertoire(s) of material culture available at a certain site in a certain period in terms of their material and stylistic characteristics’ (Versluys 2017, p. 197). The concept of the object-scape issues a powerful reminder that specific arrangements of objects introduce distinct behavioural, cognitive and adaptive constraints and promote unique possibilities of action, perception and discovery.

The inseparability of hominins and object-scapes calls attention to the conactive role of material things in the making and long-term transformation of human lifeways (*sensu* Bennett 2010). Past hominins adapted to changing material realities, but these object-worlds also scaffolded, catalysed and stipulated the behaviour of hominins, drawing them into particular pathways of action and cognition and locking-in these pathways. The inherent properties and dynamics of object-scapes and material assemblages can accelerate, propel and even re-direct transitions in the socio-cultural realm. For example, when particular things or groups of objects are no longer available, old objects become re-furbished, co-opted and/or re-interpreted, but also when entirely novel things enter human social life or object-scapes experience internal differentiation and destabilisation and seek new equilibria. The point is that changes in the pool of material things are not just the symptom of more fundamental evolutionary processes, thing-powers and assemblage dynamics bequeath historical agendas of their own, bestow path dependencies and actively configure the material conditions of human life. And yet, hardly any attention has hitherto been paid to object-centred interpretations and explanations of major patterns and trends in the Palaeolithic record. What is the contribution of material things to human deep history? Can we discern the role of material things as animators, *agents provocateurs*, game changers and perhaps even obstacles in the Palaeolithic past?

Object-centred perspectives challenge the traditional macro-archaeological rendering of long-standing palaeoarchaeological units as ‘techno-complexes’ (Clarke 1968; Gamble 2001; McNabb 2007), ‘civilisations’ (Leroi-Gourhan *et al.* 1976; Barbaza 1999; Bon 2009) or ‘leptolithic lineages’ (Laplace 1966; Marks 2003; Boëda 2005). These millennial-scale entities share some overarching features and harbour broadly similar object-worlds yet they also exhibit much neglected internal variability, spatial differentiation and gradients of chronological development. Each of them supplies unique *material conditions of existence*, illustrating the contingencies, potentialities and long-term consequences of human life in disparate object-scapes. Importantly, these unit-specific internal dynamics of stability and change—comprising cycles of formation, consolidation and decline—remain difficult to explain solely in terms of human agency or external causal factors posed by climate or biophysical environments (*e.g.* Audouze and Valentin 2010; Klaric 2013): The respective trajectories, tendencies and patterns of material culture re-organisation commonly transcend the perception and decision-making horizons of human individuals or social groups and encompass multiple generations (*cf.* Hussain and Riede 2020), suggesting that the materialities of the object-scapes themselves may represent a principal force of transformation and long-term biocultural evolution.

Rather than investigating ‘techno-complexes’, ‘civilisations’, ‘lineages’ and other higher-level units of analytical and taxonomic concern in terms of what they may *represent* and *mean*—prioritising aspects of hominin sponsorship, ‘behavioural modernity’, cosmology, kinship and types of social organisation—we should perhaps begin to interrogate them as *long-term co-productions* of human and non-human agencies and orientate the analysis towards the changing conditions of material facilitation, collaboration and closure. An object-centred perspective openly questions the privileged role of hominins in the configuration, consolidation and long-term evolution of archaeological macro-units and draws attention to the latter’s *autocatalytic* and *self-organisational* properties. It also makes room for a radically emergent understanding of the patterns in question: If we introduce the possibility that entities such as the LRJ (*Lincombien-Ranisien-Jerzmanovicien*), the MTA (*Moustérien de tradition acheuléenne*), the Initial Upper Palaeolithic (IUP), the Protoaurignacian or the Gravettian reflect varying confederacies of hominins, objects, animals and landscapes, their problematic reification may be circumvented, and they can come into view as evolving multispecies assemblages, comprising humans but also things and technologies (*sensu* Haraway 1985, 1991).

This perspective shifts the attention to the internal relationships between hominins, material things and other non-humans and underscores the equifinality of the emerging configurations: Even archaeological units of the same taxonomic order may be the result of different agencies or a dissimilar combination of agencies. It is for instance conceivable that the Châtelperronian, the Uluzzian or the IUP epitomise *multispecies edifices*—joint efforts of humans, potentially even multiple hominin forms, and various non-humans including specific object assemblages, where each implicated agent contributes distinct resources and potentials as well as modulates and constrains the options of the other agents. Interrogating these entities in terms of hominin sponsorship or cognitive capacity may then be a fundamentally misguided enterprise, obscuring rather than elucidating the multispecies and ‘more-than-human’ nature of the respective archaeological phenomena.

**Fig. 2:**
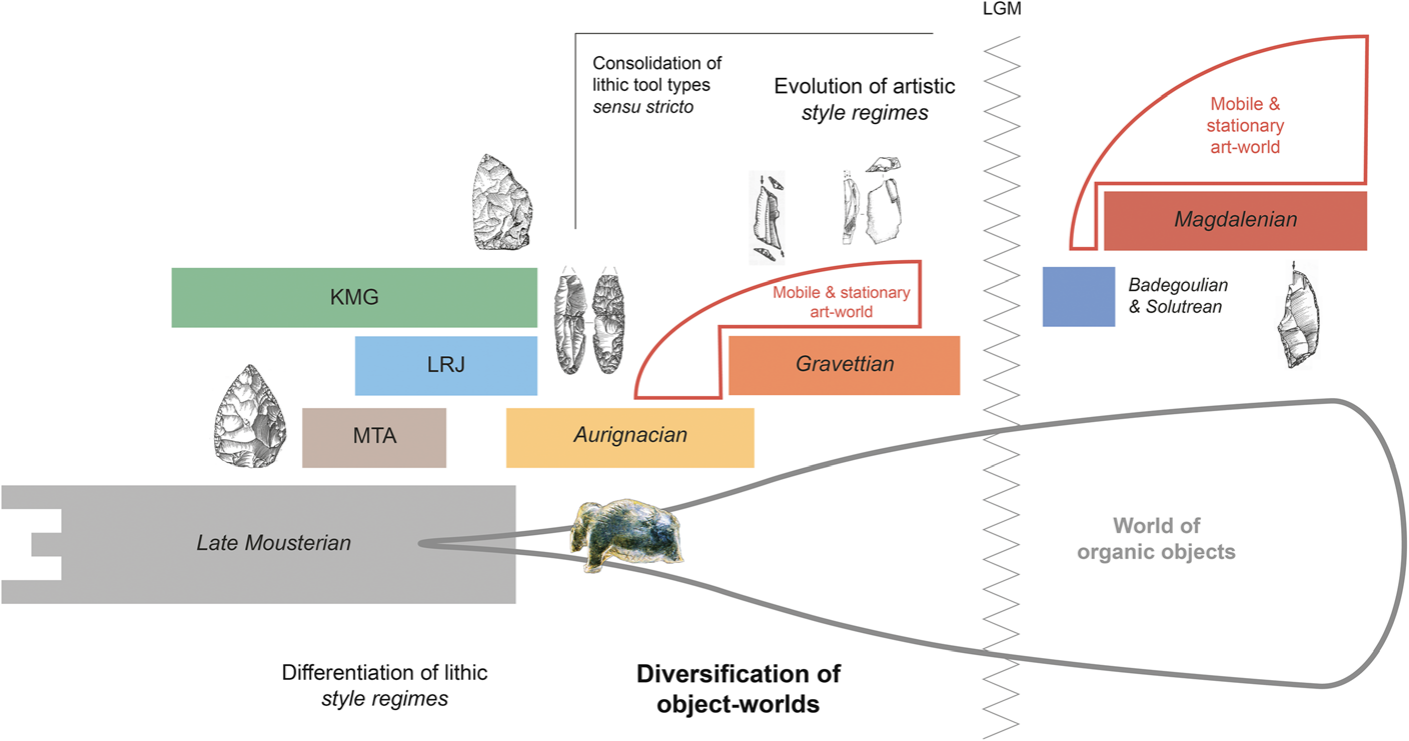
Evolution and long-term trajectories of object-scapes in the European Late Middle and Upper Palaeolithic. The Late Pleistocene heralds an increasing differentiation of object-style regimes, powering distinct and sometimes counteracting, developmental trajectories (KGM, MTA, LRJ and other techno-cultural entities; see main text for explanation). With the onset of the developed Upper Palaeolithic in Western and Central Europe, object-worlds become progressively diversified, with domestic organic tools, personal ornaments and artistic objects joining the ranks of lithic artefacts. The consolidated Upper Palaeolithic finally documents the rise of well-defined and comparatively short-lived typological systems as well as the dawn of genuine artistic style regimes

Material agency considerations also motivate a critical re-assessment of coalescing style regimes in the Late Pleistocene of Western Eurasia. From a materiality perspective, ‘style’ is not merely the antonym to function, nor is it necessarily linked to the idea of a mental template or signals human design. Instead, style comes to the fore as a potent *material effect* (Forge 1973; Coupaye 2017): Enduring style regimes have the propensity to enact long-term drawing powers, to root and re-model particular object-scapes or to entrench pre-invested developmental trajectories. In this manner, object-styles can emerge as crucial touchstones of human life and history-making, actively inducing change in other socio-material domains. Style then musters a distinct material vibrancy and becomes an evolutionary factor in its own right. A well-defined ‘geography of style’ appears to have fully developed only in the Late Pleistocene when deep-rooted developmental pathways begin to diverge and material culture repertoires considerably expand and diversify in space and time (*cf.* Langley *et al.* 2008; Hussain and Floss 2015). While temporally durable and spatially confined style regimes remain rare or hard to detect in the Lower and earlier Middle Palaeolithic, the European Upper Palaeolithic seems to have provoked a consequential shift in the significance of style, transforming it from a category largely confined to tool manufacture and perhaps *façonnage* to a wider organisational principle of blank production and the makeup of entire operational sequences (Fig. 2). From an object-centred perspective, this regime shift heralds a new *material logic* rooted in the need of generating suitable laminar blanks to supply specific tool types (projectile points and other types of lithic armature; *cf.* Pelegrin 2000, 2011; Valentin 2008; Bon 2009; Teyssandier *et al.* 2010) which continues to power long-term archaeological developments throughout the Upper Palaeolithic.

This proactive role of style may also be reflected in the advent of formalised lithic tool types during the Late Pleistocene. Foreshadowed by the individuation of increasingly diagnostic Late Middle Palaeolithic tool forms such as the triangular bifaces of the MTA, the leafpoints of the KMG (*Keilmessergruppen*) or the British *bout coupé* handaxes (*e.g.* Richter 1997; Soressi 2002), the subsequent Upper Palaeolithic is linked to the maturation, consolidation and differentiation of sequenced and often geographically specific style regimes with an evolutionary rhythm and dynamic of their own (Fig. 2). As already noted by Weissmüller (2003, p. 180–182), and recently Valentin (2011, p. 47), this formalisation of type categories from the later Middle Palaeolithic onwards points to a changing status of these objects within their object-scapes and suggests that the respective lithic artefacts acquired affective qualities, playing a key role in the definition and reproduction of the societies they helped to forge and define. In the words of Feldman (2014, p. 6), the agency of these *keystone objects* helped to ensure the perpetual ‘activation of collective memories, constituting social communities along both spatial and temporal axes’. This conactive significance of object-style in the making of Late Pleistocene societies is further expressed by the temporal dynamics of evolving lithic toolkits: Stone tool assemblages have only experienced subtle yet directed changes in their composition and structure, and phase-transitions within the classic Upper Palaeolithic periods are commonly tied to the arrival of new object forms and the gradual or sometimes sudden disappearance of other types (*e.g.* Klaric 2013). The classic sequence of burin and endscraper forms in the Western European Upper Palaeolithic is a point in case, indicating that object-styles not only fostered particular ways of life, their non-arbitrary chronological enchainment—*e.g.* the succession of *Noailles* and *Raysse* burins in the Gravettian (David 1995; Klaric 2008)—was also capable to re-configure the total space of human technical, social and aesthetic needs as well as to create previously unrecognised needs (*cf.* esp. Hodder 2011, p. 161–164, 2014, p. 20–21). Such processes initiate powerful human–object loops, which ‘feed forward’ in time (*sensu* Tomlinson 2018, p. 8) and influence trajectories of tool production and tool–technology relations. Assemblage-internal dynamics shape future possibilities and *pre-configure* outcomes (*cf.* Valentin 2008; Langlais 2010), in this way contributing actively to the formation of long-term archaeological patterns.

Palaeolithic archaeology is uniquely positioned to examine the consequences of particular object ecologies on evolutionary timescales and to chart object–object interactions escaping direct human control. A good example is the notable expansion and re-structuration of object-scapes at the Middle-to-Upper Palaeolithic junction in Central and Western Europe (Bon 2015; Hussain and Floss 2015; Floss 2018), which introduced new material possibilities, affordances and synergies but also inter-object dependencies and novel socio-material imperatives between ca. 60 and 30,000 years ago. The explosion of organic objects and technologies linked to the formation of the Early Upper Palaeolithic supplied human societies with demanding objects such as bone points, requiring constant curation, maintenance and anticipation and drawing their bearers into reciprocal investment dependency relationships (Bon 2009, p 263–268, 2015). Other object-claims can better be studied by adopting a broader comparative, transepochal perspective: Recent research has for example shown that distinct kinds of Upper Palaeolithic objects—*e.g.* stone artefacts, personal ornaments and mobile art—frequently yield *asynchronic* evolutionary trajectories (*e.g.* Audouze and Valentin 2010; Perlès 2013; Stiner 2014; Naudinot *et al.* 2017a, b), suggesting that varying object domains are prone to divergent temporal behaviours and that object evolution can be strongly dependent on concomitant transformations in wider object ecology, problematising correlational readings of these long-term changes simply ignoring object agency. Novel objects, especially those conveying, translating and hinting at formal content, provide cognitive affordances—material things are ‘good to think with’ (*e.g.* Roepstorff 2008)—and are hence capable of re-modelling human imaginative horizons, *e.g.* when assisting individuals and societies in the storage and cross-generational transformation of social information as well as in the formation and reproduction of cultural memory (Bon 2009, p. 267; Porr 2010).

From a macro-archaeological point of view, materiality theory therefore motivates at least two re-considerations of received wisdom in the field: (1) Object-centred perspectives defuse the long-standing Cartesian trope of ‘mind over matter’ reducing Palaeolithic objects to mere expressions or outputs of hominin mentalities. Taking stock of the agency of material things leads to a reversal of the equation and sheds light on human cognition as a property *contingent on* material worlds rather than presupposing them. The putative cognitive achievements of the ‘Upper Palaeolithic revolution’ (Mellars and Stringer 1989; Bar-Yosef 2002) are thus perhaps only poorly understood as a result of the newly acquired cognitive capacity of percolating *Homo sapiens* populations, but may instead be regarded as a consequence of fundamentally altered and expanded object ecologies and human–object intersections. (2) Materiality theory supplies new means to overcome the current impasse of interpreting large-scale spatiotemporal archaeological units: The Aurignacian, Gravettian and Magdalenian and other such entities can be re-described as actively evolving *material dispositions* that shaped, directed and constrained human life for thousands of years, rather than being time and again re-cast as the creations of some mysterious ‘Aurignacians’, ‘Gravettians’ or ‘Magdalenians’.

## Lithic Technology: the Three Axes of Materiality

Following on the previous perspectives on the macro-archaeological scale, this section explores alternative research avenues to past stone artefact technologies prompted by materiality thinking. Rather than offering a complete survey of the analytical and interpretive possibilities of materiality theory, deviating from or disrupting common apprehensions of the archaeological record, this section probes into a small selection of promising themes and issues profiting from materiality-oriented inquiry. We focus on three interrelated dimensions or aspects of the Palaeolithic record: (1) Geospatial, (2) technical and (3) evolutionary. The objective is to showcase the large bandwidth of ideas, concepts and perspectives supplied by object-theoretical renderings of lithic technology and their capacity to further, re-direct and, if necessary, subvert palaeoarchaeological discourses and narratives.

### The Agency of Emergent Lithoscapes

Object-oriented perspectives to the pre-Holocene human past not only take into account the dynamic ‘intra-actions’ (*sensu* Barad 2007, p. 141) of assemblages and object-scapes, they can also transform how we explore and compare the various ways in which non-human objects shape and diffract geophysical landscapes and, in this manner, *co-configure* the physicality of hominin-inhabited topographies and landforms. Spatial agglomerations of human-made objects can develop landscape-scale agencies that alter human–environment interactions and modulate or even prefigure how hominins navigated, utilised and adapted to these landscapes. Materiality theory issues a general reminder here that both geological forces and hominin behaviour can modify the uppermost band of the lithosphere in such a way that new conditions of perception, decision-making and adaptation emerge. These changing conditions of hominin landscape-dwelling play a key role in shaping millennial-scale developments in hominin land-use and mobility. The unique temporality of these dynamics suggests that the target object-mediated processes are mostly withdrawn from direct human intentionality, legislation or control, underscoring the agential quality of spatial arrangements of rocks and stones—their ability to ‘make a difference’ for human behaviour and interfere with it.

From the very moment hominins started to extract lithic raw materials from the landscape, transform these materials, carry them around and discard them, they began to affect and manipulate the structure of the lithosphere (Pope *et al.* 2006; Pope 2018). Even though the details and scale of this impact vary in time and space, it resulted in the re-distribution of stone resources and lithic objects in the landscape, potentially re-modelling subsistence and mobility affordances, and perhaps even erosion and vegetation patterns. Foley and Lahr (2015) have documented such object-mediated landscape transformation in the Messak Settafet—a large sandstone massif in the Libyan Central Sahara. Over a period of at least 500,000 years, hominin stone working activities have created a large and dense anthropic environment formed by lithic artefacts and debris. It has been argued that this massive lithic-strewn pavement—stretching a total length of over 350 km and averages about 75 artefacts per square meter—is the product of repeated hominin visits and reflects one of the earliest examples of human ‘landscaping’, the time-transgressive built-up of novel environments through recurrent hominin activities at specific locales (Foley and Lahr 2015).

**Fig. 3:**
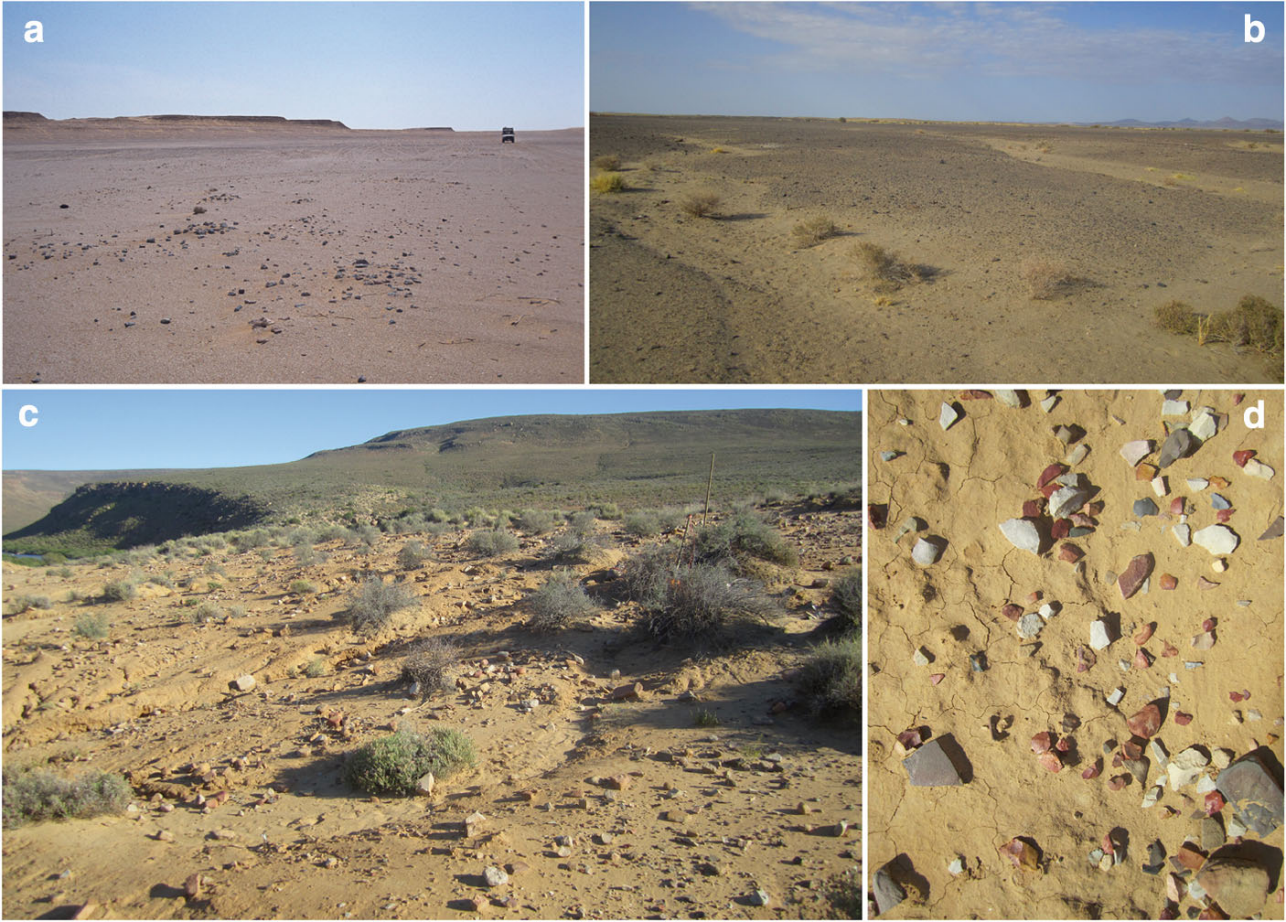
Examples of stone artefact pavements: **a** Palimpsest surface locality 83/11, Sitra, Egypt, possibly spanning the Late Pleistocene and the Early to Mid-Holocene (Cziesla 1989); **b** palimpsest surface of Middle and Late Pleistocene stone artefacts separated by erosional gullies in West Turkana, Kenya; **c** surface of predominantly Middle Stone Age (MSA) lithic artefacts resulting from the recent erosion of river terraces in the Doring River catchment at Uitspankraal 7, South Africa; **d** close up of stone artefacts from the surface at Uitspankraal 7, South Africa, where several refits were achieved. Photo credits: **a** AAArC—African Archaeology Archive Cologne: ID 5406662 (https://arachne.dainst.org/entity/5054557), © Rudolph Kuper; **b**–**d** © Manuel Will

Yet, the story of the Messak Settafet is not just about hominins. The Acheulean and Middle Stone Age (MSA) artefacts composing this compact ‘veil of stones’—borrowing an idiom by Isaac (1986)—also acted as an external raw material reservoir for mobile hominin populations living in the Pleistocene Central Sahara. Regardless of whether or not the Messak Settafet represents a deliberately engineered landscape, the large accumulation of workable stone created a focal point in geographic space, anchoring a broad range of hominin activities by influencing mobility and provisioning decisions. The Messak Settafet is a prototypical ‘lithoscape’—a palimpsest landscape constructed and furnished with lithics—which invites stone foraging trips and emerges as an artificial raw material outcrop and potential (re-)tooling cache. As part of the external material environment of early North African hominins, the locality exemplifies landscape-scale human–thing dialectics over many thousands of years and provides evidence of the geographic agency of object assemblages—their ability to channel and reinforce spatial patterns of hominin behaviour and lithic technological organisation (Fig. 3).

**Fig. 4:**
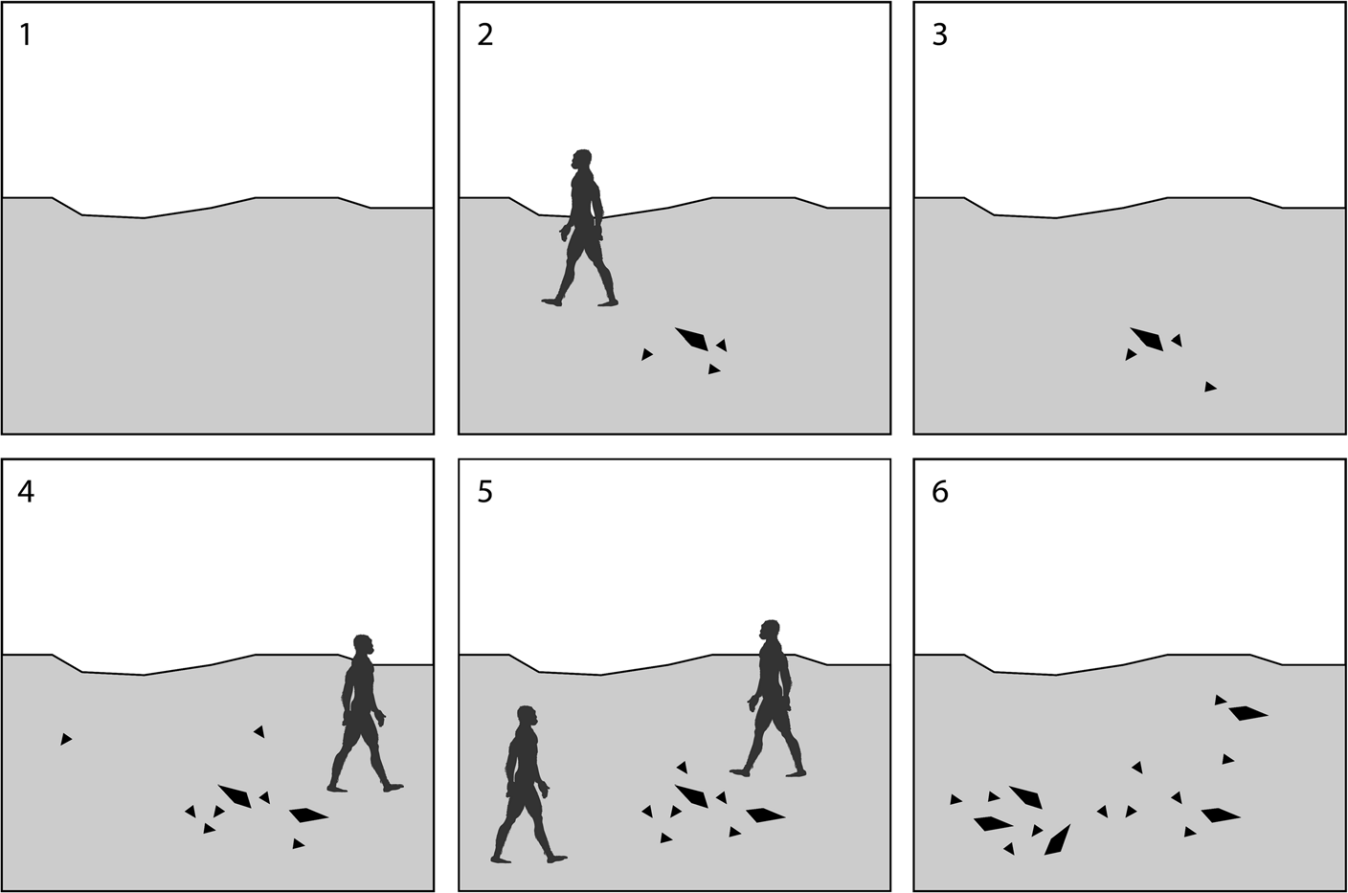
Schematic illustration of the possible build-up of an emergent palimpsest lithoscape, acting as a future material attractor for hominins in the wider landscape. Numbers are arranged in chronological sequence; dark grey humanoid outlines represent incoming and outgoing hominins and black angular icons denote accumulated lithic items (small icons: small *débitage* and flakes; large icons: LTCs, large manuports, *etc*.). Note that the probability of exploiting and accumulating stone artefact material and debris generally tends to increase as a function of the total synchronic and diachronic intensity and frequency of hominin–lithoscape interaction

The Palaeolithic record indicates that the Messak Settafet is no isolated case: Lithoscapes have played an important role in the evolution and expansion of the ‘human niche’ across the entire globe (Hiscock 2014; Pope 2018, p. 36f; Stout *et al.* 2019), transforming the material ecology of early hominins and increasing the availability and predictability of stone in their environments (Pope and Roberts 2005; Pope 2018). These lithoscapes do not merely *record* or *testify to* past hominin activity; they have conactively *shaped* this behaviour and their materiality is a structure-giving element of long-term socioeconomic strategies and landscape perceptions. The large cutting tool (LCT) phenomenon illustrates this active involvement of diversified lithic environments in the making of the Palaeolithic record. LCTs—heavy and often bulky stone artefacts such as cleavers, handaxes and other large *façonnage*-tools—exhibit structured use, transport and discard patterns (Isaac 1980; Clark 1987; Villa 1990; Ashton *et al.* 1998; Soriano 2000; Dennell 2018b) and often simultaneously serve as tools and reduction matrices (*cf.* Pope and Roberts 2005, p. 82). LCT design not only has an instrumental dimension, but also renders these artefacts attractive units of extraction for already preselected and tested raw materials. Through the lens of material agency, the growing importance of and increasing reliance on LCTs during the Middle Pleistocene may not only signal a generalised shift in hominin subsistence and foraging patterns, but also reveal a snowballing evolutionary trajectory of *adapting to self-generated material worlds* and coping with the attendant constraints and possibilities. LCT-rich archaeological assemblages from North-Western Europe—*e.g.* those encountered at Boxgrove, High Lodge, Cagny and Soucy (Pope 2002; Nicoud 2011) but also at sites in Africa and the Near East, such as EF-HR (de la Torre *et al.* 2018), Isenya (Roche *et al.* 1988) or Gesher Benot Ya’aqov (Goren-Inbar *et al.* 2016)—may then be re-interpreted as special locales tied to the provision of diverse raw materials, flexible preforms and readily deployable or recycable cutting edges. In other words, lithoscapes act as landscape ‘attractors’ entrenching their own position in the wider land-use system through repeated hominin visits and material input over time (*cf.* Potts *et al.* 1999; Fig. 4) and increasingly favour ‘embedded’ procurement strategies (*sensu* Binford 1979; Nelson 1991, p. 64) which depend on previous hominin behaviour rather than natural lithologies.

This conactive understanding of the relationship between object geographies and hominin behaviour is supported by detailed techno-functional analyses of LCTs (*e.g.* Soriano 2000; Nicoud 2011, 2013) and the presence of double-patinated bifaces and other heavy-duty tools in Lower Palaeolithic or earlier Middle Palaeolithic sites (Soriano 2000). McPherron’s (1994) meta-analysis of biface-bearing assemblages from Central and North-Western Europe similarly lends support to the lithoscape hypothesis: His data indicate that the reduction intensity of bifaces is positively correlated with the total number of these artefacts occurring in a given assemblage, suggesting that larger biface assemblages tend to document more advanced transformation stages (see also McPherron 1999, 2003). This observation fits the idea of behavioural reinforcement through lithoscapes, which modulate the distribution, exploitation modalities and life-history trajectories of the partaking stone artefacts.

That LCTs are often missing from smaller archaeological sites or surface scatters is then perhaps not only an expression of the heightened mobility of these artefacts (Clark 1987, p. 809; Kelly 1988; Soriano 2000; Turq *et al.* 2013), but may bespeak of the emergent, long-term consequences of hominin–landscape co-evolution and niche construction (*cf.* Riede 2019; see below). For example, the duality of biface-rich open-air sites and biface-poor cave assemblages in the Chinese Lower Palaeolithic may not merely reflect the task-specific organisation of hominin land-use (Hou *et al.* 2000; Wang 2005; Li *et al.* 2014; Wang *et al.* 2014; Yang *et al.* 2014; Dennell 2018a, p. 272, 2018b), it may similarly signal the active role of bifaces and other large lithic objects in the *construction* of differentiated locales with affordances and behavioural significances deviating from cave settings. This geographic agency of the lithoscape is also evident in archaeological simulation and agent-based modelling (ABM) applications (esp. Haas 2014; Davies and Holdaway 2017; Haas and Kuhn 2019; Holdaway and Davies 2019). Investigating the material effects of lithoscapes hence promises to overcome the received view that gradually built-up object landscapes with their palimpsest, emergent and time-averaging qualities (Stern 1993, 2008; Wandsnider 2008) inhibit insight and interpretation (*cf.* Dibble *et al.* 2017, p. 825–833; but see Bailey and Galanidou 2009). In a similar vein as ‘techno-complexes’, ‘technical currents’ (Valentin 2011, p. 56–59) or ‘lineages’, some place-making object assemblages are perhaps better understood as millennial-scale co-creations of humans and non-humans, even implicating multiple hominin populations and taxa. Although controversial, this emerging view is supported by recent ABM findings, for example by the seminal study of Coco *et al.* (2020) demonstrating that ‘transitional’ stone artefact industries may be by-products of the long-term re-working, re-cycling and scavenging of pre-furnished lithoscapes.

Materiality theory also re-vitalises classic debates on taphonomy and the role of varying landscapes and environments in human evolution and promotes fresh research angles on hominin–thing–landscape constellations. Thing-powers, assemblage agencies and the affordances of objects often depend on how things intersect with broader geophysical landscape characteristics (*e.g.* Kourampas 2012). For example, in open and relatively arid landscapes where sedimentation and object-burial are thwarted, lithoscapes are more likely to develop autonomous agencies as erosion quickly exposes lithic objects and surface accumulations are easier to spot from afar. The erosional and fluvial accumulation of raw materials and lithic artefacts in natural gullies and depressions may further pre-establish object-invested landscape structures, which may subsequently attract hominins and invite additional material input, artefact production and object re-working. This effect can be substantially reinforced if such conditions occur in strategic landscape positions, *e.g.* at the interface between different landforms, elevational belts or ecozones as proposed for some Oldowan assemblages in southern Kenya (Potts *et al.* 1999). Palimpsest landscapes—whether anthropic, geogenic or hybrid—often provide unique material clues and affordances which open up or close specific trajectories of material culture evolution, scaffold social learning (Tostevin 2019) and promote material *bricolage* (*sensu* Lévi-Strauss 1966 [1962], p. 17–21; *cf.* Duymedijan and Rüling 2010; see below).

### The Vibrancy of Lithic Technology

Object-centred approaches call attention to the technology-internal consequences of past material configurations. Instead of wrestling for the precedence of social, environmental or technical factors for explaining the human deep past, materiality and object-oriented approaches insist on irreducible human–thing confederacies and the perpetual co-construction of hominins and technologies throughout the Palaeolithic (*cf.* Hussain 2018b). The materiality prism undercuts problematic Cartesian polarities and seeks to transcend the enduring preoccupation with technology as a ‘static’, ‘inert’ or even ‘passive’ human product. Albeit undeniably representing material productions, stone artefact technologies are also generative, producing agents in their own right. This renewed perspective on lithic technology sheds new light on foundational debates in lithic studies, defuses long-standing certainties and offers a means to integrate socio-cultural and technoanthropolgical research trajectories (*e.g.* Carbonell and Mosquera 2006; Bon 2009; Pelegrin 2013) with the insights of ecological (*e.g.* Binford and Binford 1968; Kelly 1988, 2014; Nelson 1991; Kuhn 1995) and reduction theory-driven lithic analysis (*e.g.* Dibble 1987; Holdaway 1991; McPherron 1994).

**Fig. 5:**
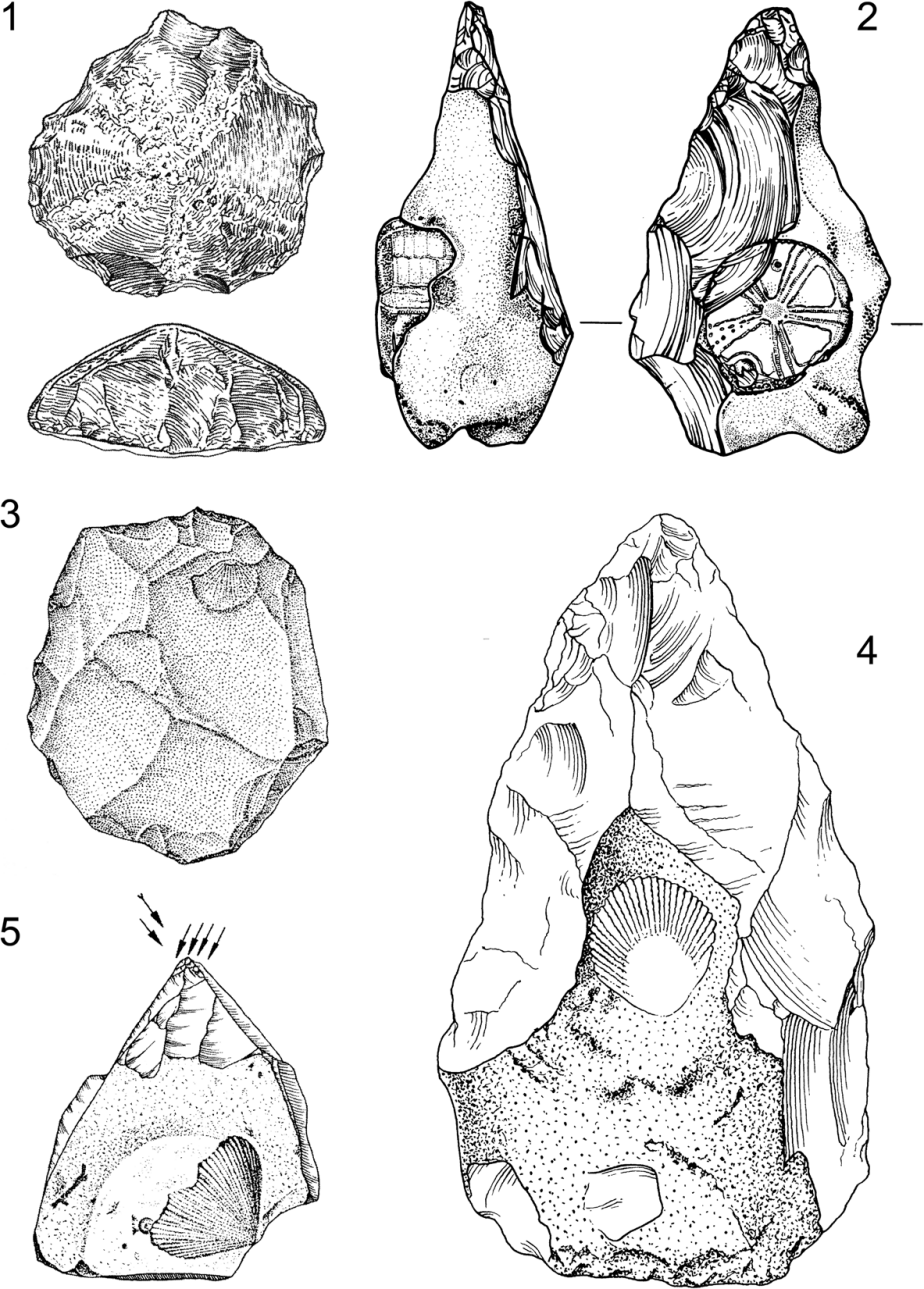
Selected stone artefacts whose production and design responds to raw material configurations, properties and physical idiosyncrasies: (1) Circular scraper from the Acheulean of Saint-Just-des-Marais (Oise, France) manufactured on a Cretaceous fossil sea urchin (Oakley 1971); (2) pointed Lower/early Middle Palaeolithic handaxe from Swanscombe (Kent, England) bearing a fossil sea urchin inclusion (Oakley 1981: Figure 3); (3) Mousterian quartzite scraper from Schweinskopf-Karmelenberg (Mayen-Koblenz, Germany) with a brachiopod imprint (Schäfter 1996); (4) Lower Palaeolithic handaxe from West Tofts (Norfolk England) with a bivalve shell at its centre (Oakley 1981: Figure 2); (5) large Upper Palaeolithic burin (possibly Aurignacian) with fossil shell inclusion (Lorblanchet and Bahn 2017: Figure 7). Objects not scaled

Students of the Palaeolithic are long aware that the *Gestalt* and configurational properties of stone raw materials can be important factors influencing the makeup and organisation of lithic technology (*e.g.* Turq 2005; Browne and Wilson 2011; Moncel and Daujeard 2012; Fernandes *et al.* 2013; Raynal *et al.* 2013). Textbook examples such as the bifaces from Swanscombe and West Tofts in Britain (Oakley 1981; Lorblanchet 1999, p. 89–90) or the Mousterian quartzite scraper from Schweinskopf-Karmelenberg in Germany (Schäfer 1996) suggest that raw material saliences—especially geological inclusions such as fossils—have at least occasionally played a decisive role in the conception, manufacture and design of Palaeolithic stone artefacts (Lorblanchet and Bahn 2017, p. 50–56; Fig. 5). Raw material studies similarly underscore the prevalence of specific material–technology co-dependencies, often embedded in distinct trajectories of socio-material change (Floss 1994; Féblot-Augustins 1997). The articulation of Upper Palaeolithic volumetric blade extraction strategies with Bergeracois flint in southwestern France (Demars 1989, 1994) or the interweaving of quartz and bipolar knapping throughout much of the Pleistocene, rooted in the former’s non-conchoidal flaking properties (Knight 1991; Driscoll 2011; Pargeter and Hampson 2019), are classic examples of this bond. The frequently encountered raw material dichotomy between LCTs and small tools at Lower and Middle Palaeolithic sites also suggests that the material quality of workable stone was an important determinant of the past (*cf.* Bar-Yosef and Goren-Inbar 1993; Sharon 2007; Harmand 2009). In addition, techno-economic research has produced a wealth of evidence for the non-arbitrary coupling of knapping modalities, reduction trajectories, object biographies and artefact mobilities with specific raw material affordances and possibilities (Perlès 1980, 1992; Geneste 1985; Boëda 1991; Renard and Geneste 2006; Kuhn 2011). Re-assessing these and other cases of human–material–technology intersection from the perspective of materiality theory helps to avoid the pitfalls of economic or cultural determinism and to map the conactive linkage and changing power relations between the involved human and non-human agents.

Late Magdalenian blade production in Western Europe illustrates the capacity of materiality theory to break up and subvert the raw material discourse. Magdalenian blade extraction systems with their elevated preparatory investment, blank productivity and reliance on fine-grained, high-quality raw materials (*cf.* Pelegrin 2011) are either regarded as sophisticated and efficient adaptations to the rich and diversified lithology of North-Western Europe or interpreted as a specific technical choice tied to the subsistence, mobility and social networking of their bearers (Jochim 1991; Straus 1996, p. 95–96; Féblot-Augustins 2009). Materiality theory calls these readings into question and inverts the logic of analysis: The close tie between a highly invested laminar reduction strategy and raw materials of exceptional quality may equally be explained as a long-term consequence of the specific *trade-offs* and *requirements* of adopting the complex and over-determined Magdalenian blade reduction system (*cf.* Bon 2009, p. 252; Langlais 2010, p. 301). In this view, the dependence on high-quality stone raw materials is considered a *side effect* of optimising, integrating and rigidifying the volumetric exploitation of standardised blades, rather than a primary human preference. While de-centring the human, this account also draws attention to the potential of evolving technologies to place increasingly strict *demands* on their users. The functionality of such technologies comes at a price and entraps people into a material logic largely dictated by technologies. Long-term socio-material developments leading to the endorsement of costly technologies and objects can in this way emerge as co-determinants of human history and fundamentally re-shuffle the power relations between humans, raw materials and lithic technologies. The advantages of specialised blank production systems conceal the dark side of accruing human–technology confederacies: The gradual *enslavement* of humans by increasingly adamant and self-contained technical worlds (*cf.* Cooper 1995). Late Magdalenian blade production can then be re-assessed as a difference-making force on its own, shaping and entrenching the course of Late Upper Palaeolithic evolution, rather than merely ‘flowing out’ of human cultural history.

This systemic agency of lithic technology helps to elucidate the internal structure of larger technical worlds and to work out the ‘intra-active’ dynamics (*sensu* Barad 2007) which stabilise, restrain and propel them. The material vibrancy of lithic technology fosters and prefigures behavioural pathways, creates human–thing co-dependencies and catalyses the gradual integration of varying domains of material culture and hominin biology and behaviour. The marked technical diversity of the Western European Middle Palaeolithic provides a telling example here. The period is characterised by multiple co-existing technical systems, comprising well-defined core-blank systems commonly qualified as ‘Levallois’ (Boëda 1988, 1994; Van Peer 1992, p. 1–8, 15–33), ‘Quina’ (Bourguignon 1996, 1997) and ‘Discoid’ (Boëda 1993; Mourre 2003; Thiébaut 2013) but also various laminar (*e.g.* Moncel and Daujeard 2012) and less-formalised *débitage* systems (*cf.* Delagnes 1993, 2010; Faivre 2008) with or without bifacial or lamellar components (Soressi 2002; Faivre 2008; Delagnes and Rendu 2011; Brenet *et al.* 2014). This complex technological infrastructure demonstrates hominin action and design, but it also carries a legacy of its own and conactively moulds key dimensions of hominin behaviour such as mobility, raw material economy and blank selection. The partaking technological systems enact unique techno-economic trade-offs, affording disparate tool conceptions, promoting varying degrees of blank normalisation and productivity, as well as creating differential possibilities for blank transformation and depletion (Bourguignon *et al.* 2006; Delagnes and Rendu 2011; Brenet *et al.* 2014). Different technical systems also support—and to some extent *enforce*—divergent rates of structural ‘ramification’ (Geneste 1991; Tixier and Turq 1999; Bourguignon *et al.* 2004, 2006) and greatly vary in terms of technical investment and flexibility (*cf.* Meignen *et al.* 2009).

The overarching raw material tendencies and preferences of varying Middle Palaeolithic technical systems may therefore only partially explained in terms of human intentionality, ingenuity and planning and are also dictated by the requirements and material consequences of the respective technical systems themselves. Middle Palaeolithic ‘technicity’ (*sensu* Simondon 1958; Stiegler 1994)—the inseparability between hominins, artefacts and the wider technical world—encourages the specialisation of lithic systems and the infrastructural division of labour, promoting diversified system-level ‘performance characteristics’ (*cf.* Schiffer 2011, p. 26–28) and fostering specific technology–raw material confederacies (Geneste 1985, 1989; Meignen *et al.* 2009). Preferential Levallois methods, for instance, uniquely profit from high-quality raw materials and justify exceptional raw material and preparatory investment (Boëda 1993; Delagnes and Meignen 2006), while Quina methods take advantage of rectangular nodules, and afford thick, naturally backed tools with triangular cross-sections and prolonged reworking cycles (Turq 1989, 1992; Bourguignon 1997; Hiscock *et al.* 2009). Discoid reduction methods, by contrast, relax raw material dependencies and may be deployed flexibly in order to derive a heterogeneous range of blanks and cutting-edge configurations, even from lower-quality raw materials (Boëda 1995; Slimak 2003). These intra-active system dynamics have repercussions for the portability of cores, blanks and tools (Delagnes and Rendu 2011; Turq *et al.* 2013) yet also determine the possibilities of complementarity and cooperation between varying lithic systems (*cf.* Soriano 2000), and thus frame the horizon of potentialities and constraints faced by the hominins using and relying on these technologies.

**Fig. 6:**
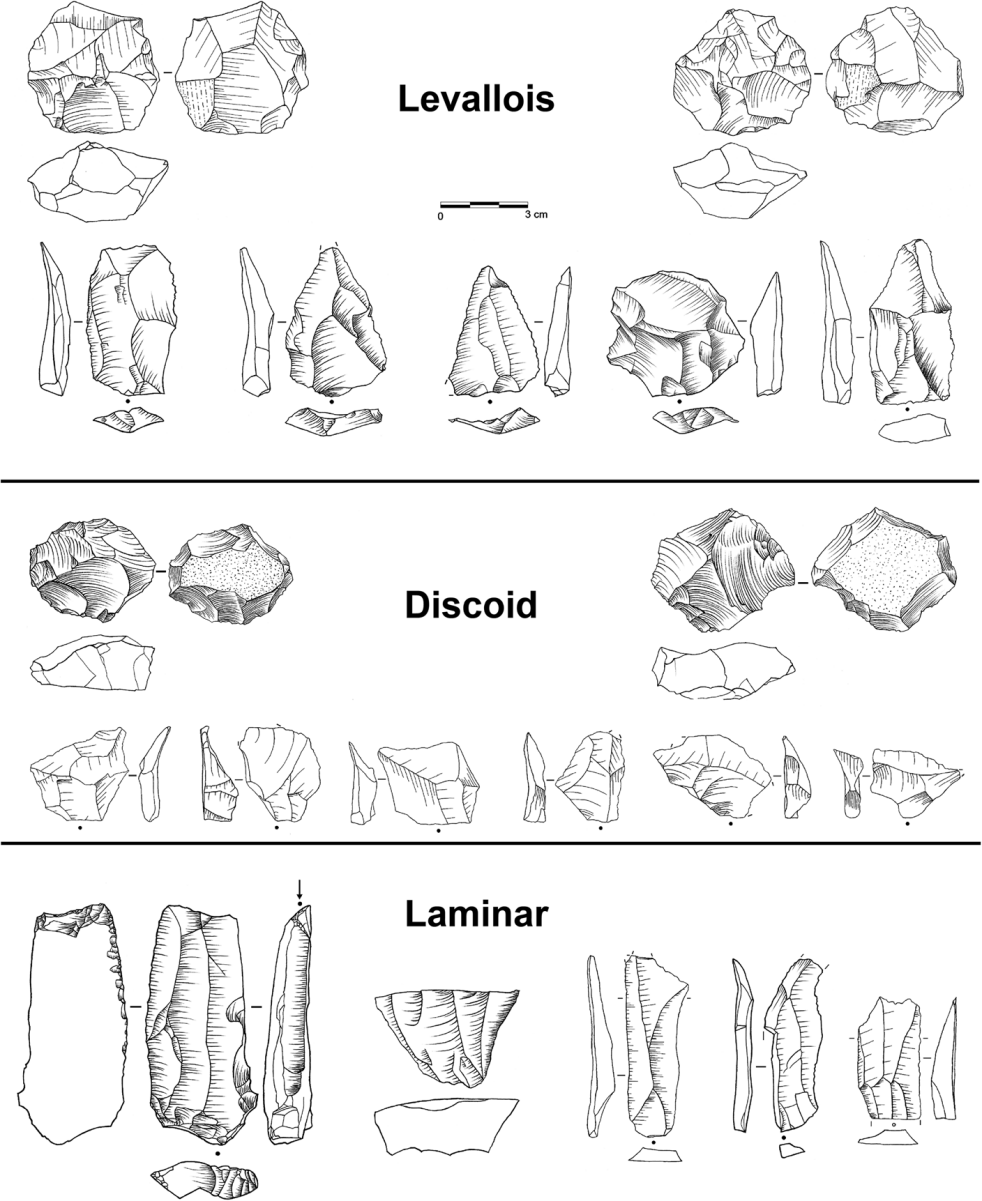
Lithic technological variability in the Middle Stone Age (MSA) layer ‘POX’ of Sibudu (South Africa) dated to ca. 58 ka BP and part of the larger ‘Sibudan’ techno-complex. Note the synchronic co-occurrence of various well-defined technological strategies and conceptions with some of their associated cores and blanks

A similar case can be made for the technological infrastructure of the African MSA where differentiated technical systems regularly co-occur and complement each other (*cf.* Tryon and Faith 2013; Will *et al.* 2014; Will and Conard 2018; Fig. 6). ‘Mode 3’ material worlds (*sensu* Clark 1969) generally seem to revolve around what Arthur (2009) has called ‘structural deepening’—the tendency of larger technological systems to counteract the limitations of a single main principle of operation by promoting the coalescence of various sub-systems that take over specialised tasks. Even though representing purposed systems, different lithic technologies can thus scaffold each other’s performance characteristics and afford or delegate functions and tasks to each other. In this way, the materiality of technology emerges as a key factor in the *self-assembly* of diversified technical worlds. This organisational dimension of material worlds calls attention to the deep entanglement and ongoing co-adjustment of hominins, materials, skills and concepts of stone working, portraying technology as a proactive rather than static force in human evolution (*cf.* Hodder 2012; Leonardi *et al.* 2013).

Materiality explorations also help to refine our grasp on object use and tool functionality, underscoring that ‘objects’, ‘artefacts’, ‘instruments’ and ‘tools’ should be distinguished categorically and analytically (Rabardel 1995; Guchet 2018). Object-oriented research motivates a vocal critique of simple equations between lithic tools and stone artefacts bearing some form of deliberate edge modification (Boëda 1997; Soriano 2000), thus undermining the long-standing interpretive dualism between blanks and retouched artefacts in Palaeolithic stone artefact analysis. The thrust of the critique is also directed against traditional use-wear and functional studies—sometimes labelled ‘traceology’—which tend to focus on the *realised* diagnostics of tool-use with the consequence of ‘isolating tool finalities from the morphology and structure of lithic tools’ (Soriano 2000, p. 7–8). From an object-theoretical perspective, tool functionalities cannot be divorced from the physicality, structure and operation of objects-in-use, and tool functionality is always a question of globalised object designs, affordances and materialities, explicitly allowing for the possibility that unretouched objects equally dispose of functional efficacy and may reasonably be examined as potential tools.

The techno-functional approach in French techno-anthropology (Lepot 1992/1993; Forestier 2000, 2010; Boëda 1997, 2001, 2013; Bonilauri 2010; Donnart 2010; Chevrier 2012) with its emphasis on the character and interplay of varying techno-functional units (UTF; *unités techno-fonctionelles*) on a single technical object illustrates some of the opportunities: UTF analysis subverts traditional apprehensions of the ‘tool’ and shifts attention from realised traces and residues on artefacts to the *design determinants* and *possibilities* of lithic object use. The result is the re-negotiation of relationships between lithic objects, artefacts and tools and the investigation of their instrumental qualities (Forestier and Boëda 2018). The functionality of any given lithic object depends on the type (*e.g.* active, passive or transmissive), location and structural arrangement of its UTFs and the affording qualities of the total configuration of surfaces and edges. UTFs can be identified based on surface convexities as well as edge outlines, angles and morphologies (Albrecht and Müller-Beck 1988; Soriano 2000: Annexe 2), and their character and articulation can be compared between objects, so that a large spectrum of functional tool classes between ‘mono’ and ‘multi-tools’ grounded in specific material properties becomes tangible (Nicoud 2011; Chevrier 2012; Boëda 2013). The functionality of a lithic object and how it can be instrumentalised is considered a question of the object’s global material makeup and depends on the potentials afforded by its structure, shape and working-edge configurations. This material agency of stone tools elucidates the co-constitutive ties between matter, material design and function (Hussain 2018b) and helps to integrate high-resolution data on object-shape and geometry derived from 3D recording techniques (*cf. e.g.* Viallet 2019).

An important implication of this perspective is that not all material effects which come into view through UTF analysis need to be understood as anticipated or strictly intended by hominin stone workers. Some of these material effects may be natural, by-product or emergent outcome of other goal-directed stone working activities. The experimental character and complexity of lithic reduction (*sensu* Weissmüller 1995) continuously creates novel material affordances and design possibilities as the process of stone transformation proceeds. Not all of these actionable options are strictly engineered by the knapper, some can be described as *emergent*—they are suggested, offered and realised *within* processes of interaction and coordination between hominins, objects and materials. As shown by Boëda (1995) in the context of recurrent Levallois, these distributed material dynamics of stone working may pull and ultimately entrap hominins into distinct pathways of reduction. Materiality theory has therefore important ramifications for the contentious debate on the role of intentionality in explaining lithic technologies (*cf.* Sandgathe 2004; Dibble and McPherron 2006; Bon 2009, p. 137–138; Perlès 2016; Dibble *et al.* 2017). An object-centred perspective paves the way to a balanced middle ground between an over-intentionalised reading of technical realities (Pelegrin 1990, 2005; Perlès 1991a, 1992; Inizan *et al.* 1995) and an overly fluid, responsive and hyperplastic view of technology (Henry 1989; Shott *et al.* 2011, p. 320; Shott 2017). The conactive approach throws light on the formative hominin–thing nexus—the continuous co-making of the human and the technical—suggesting that material design is not always reducible to human will or fully controlled by it. Although lithic artefacts are by definition human-made, not every technical decision, detail or reduction step within an operational sequence must be interpreted as planned or hominin-initiated.

**Fig. 7:**
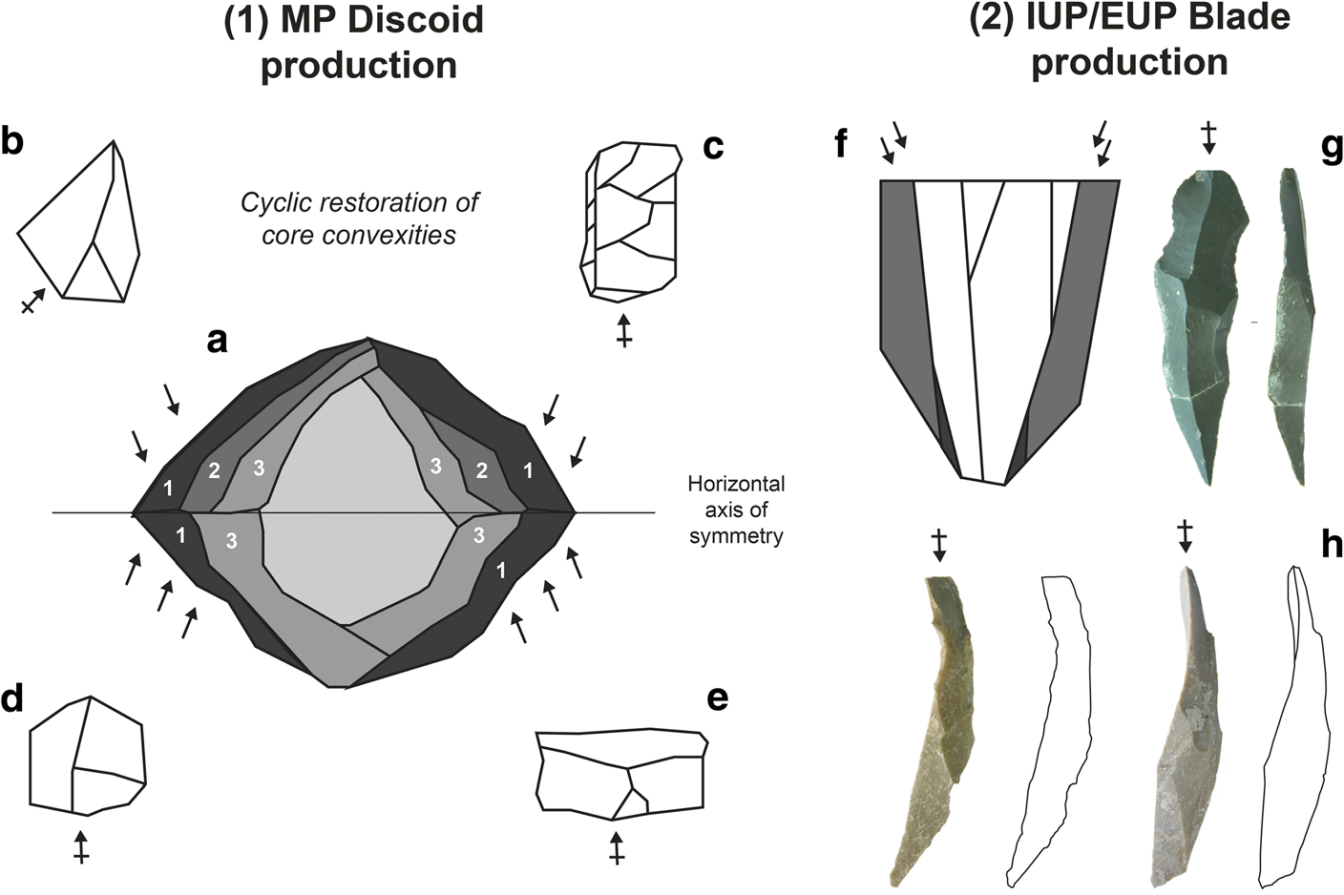
Technological relationships between volume conception, material affordances and preparatory procedures of selected lithic technologies: **a** Volume management of Mousterian Discoid production involving the cyclic (auto-)restoration of the core architecture and its corresponding blank types (**b**–**e**; re-drawn after Boëda 1993, 1995); **b** pseudo-Levallois point (cordial Discoid extraction axis); **c** *débordant* flake (cordial Discoid extraction axis); **d** rectangular flake (centripetal Discoid extraction axis); **e** broad, transverse flake (centripetal Discoid extraction axis); **f** generalised reduction scheme of selected Initial (IUP) and Early Upper Palaeolithic (EUP) blade production systems from Eurasia; **g** twisted core-edge blade used to manage lateral convexities and the distal convergence of laminar cores from the IUP of Kara-Bom (Altai, Russia; Zwyns 2012: Figure 172); **h** twisted, off-axis overshot blades used to control the lateral and distal core convexities in the Early Ahmarian of Al-Ansab 1 (Petra Area, Southern Jordan), testifying to an ‘embedded’ mode of laminar maintenance and preparation (Hussain 2015a: Fig. VII-8). Image (**g**) with courtesy of Nicolas Zwyns and (**h**) © Shumon Hussain

This co-constitution of hominins and technologies is also spotlighted by *self-organisational* properties of lithic core-blank systems, for example expressed in the modalities and logics of core preparation and maintenance. Preparatory procedures aimed at sustaining a given core-blank system may be categorised as ‘explicit’, ‘embedded’ or ‘automated’:

1. *Explicit preparation* qualifies well-defined technical operations differing from the modalities of primary production in terms of the resulting products, knapping techniques or gestures. This mode of preparation creates easily identifiable, highly diagnostic stigmata and lithic artefacts such as prepared core edges, crested blades or thick core-flank negatives.
2. *Embedded preparation* sustains the exploitation trajectory without changing much of the employed techniques, knapping gestures or artefactual output. This mode of preparation is *e.g.* exemplified by fully laminar reduction systems in which lateral, off-axis blades with minimal distal overshot ensure the constant restoration of lateral and frontal core convexities (*cf.* Blaser *et al.* 2012; Zwyns 2012, p. 219–227; Hussain 2015a; Fig. 7).
3. *Automated preparation*—or ‘auto-preparation’—denotes technical procedures which most often leave no diagnostic stigmata yet ensure the ongoing restoration of the essential reduction conditions through synergistic interactions of ordinary blank extraction operations. A chief example of an auto-preparatory technical system is provided by Discoid core reduction, where recurrent removals of circumferential blanks aim to re-furbish the semi- or bi-hemispheric convexity structure of the exploited volume (*cf.* Boëda 1993, 1995; Fig. 7). Large and globally prepared, bidirectionally exploited blade-cores from the late Magdalenian, in which the alternating extraction of overlapping blades automatically restores the frontal convexities of the exploitation surface (*cf.* Pigeot 1987; Bodu 1994; Pelegrin 2011), issue another example of self-organising core reduction.

The comparison of these preparation modalities and their articulation with the structure and materiality of their technical systems suggests that technical intentionality is often *distributed* within the operational sequence and across different technical actions, and power relations between hominins and technologies can *shift* over the course of ongoing lithic reduction. Embedded and especially automated preparation bespeaks of the power and gravity of technology-internal material dynamics, reflecting technical solutions which are variously promoted and pre-configured but also inhibited by the physical transformation patterns of the exploited core volumes. Explicit preparation, conversely, *breaks up* these material affordances and imposes new rules and exploitation structures by overwriting and disrupting object-specific dynamics, so that the involved power relations favour the hominin side of the spectrum. Similar questions can be posed with regard to general patterns of blank–tool interaction within a single technological system, for example studying whether blank production foreshadows blank selection and tool manufacture or whether blank choice and tool design are instead decoupled from blank production strategies (*cf.* Perlès 1991b; Geneste 1991; Hussain 2015b).

### The Audacity of Technological Evolution

Materiality theory not only deepens our understanding of geospatial and technical dimensions of the lithic past, it can similarly inform, strengthen and extend apprehensions of long-term trajectories of material culture change. Due to its peerless temporal coverage and low chronological granularity, Palaeolithic archaeology finds itself in a unique position to refine and perhaps revolutionise the study of technological dynamics over many millennia (Boëda 2005, 2013; Valentin 2008, 2011; Kelly 2016; Clark and Barton 2017; Perreault 2019). Hence, Palaeolithic research promises to contribute to key issues in the humanities and social sciences, such as the debate on the relationship between technical pathways of change and coercive climatic and environmental transformation in human history (*cf.* Hussain and Riede 2020). Moreover, there is a material ‘audacity’ of technological developments to expose—a ‘resistant force’ in the words of Bennett (2010, p. 1)—which becomes most tangible on temporalities exceeding traditional cultural, sociological or historical ‘shallow-time’ analysis. The perspectives and resources supplied by materiality theory can thus help to emancipate ourselves from narratives and explanatory models originally developed to address short-term phenomena (from scales of seconds to decades) or at best processes of the *longue durée* (centenary-scales; *sensu* Braudel 1949). Through the prism of materiality, we can start investigating and comparing long-term patterns in lithic technology as human–material *co-productions* obeying to evolutionary principles and mechanism that have hitherto slipped scholarly attention precisely because of their unique temporality (*e.g.* Currie 2019).

Object-centred approaches to deep-time constellations of lithic technology promote the integration of *object ecology* and *object evolution*. While material environments enable, refract and constrain the evolution of object assemblages, evolutionary forces also alter, modify and subvert the material logic imposed by these worlds. At the root of this long-term dynamic is the ‘audacity’ of hominin-inhabited material worlds: These worlds are resilient and sturdy and appear less perishable than the biocultural existences and social ambitions of their hominin counterparts. Whether intact, broken or transformed, many objects outlive their makers and may in principle be re-used, repaired and/or re-furbished within and across hominin groups or generations (see esp. Bon 2009, p. 265; Hodder 2014). Material things and technologies not only help to anchor, coordinate and reproduce social life (Pfaffenberger 1992; Cresswell 1994; Lemonnier 2014), they are passed on—consciously or not—to future generations and thereby engender a historical legacy of their own. They *pre-furnish* material ecologies, shape the possibilities of development and adaptation therein and enframe distinct ‘regimes of practice’ (*cf.* Thévenot 2001), so that hominin futures always depend on their object pasts (Stiegler 1994).

The difference-making propensity of material environments within long-term evolutionary processes is also foregrounded by niche construction theory (NCT) developed under the umbrella of the EES (*cf.* Pigliucci and Müller 2010; Laland *et al.* 2015). NCT draws attention to the modulating role of ‘ecological inheritance’ as a third inheritance system in the evolution of living organisms (Day *et al.* 2003; Odling-Smee *et al.* 2003; Odling-Smee 2010)—next to genetic and cultural inheritance—and has recently made an effort to articulate the repercussions of specific pathways of *material inheritance* (Ellis 2015), re-configuring ‘fitness landscapes’ (*sensu* Wright 1931) and evolutionary trade-offs. While materiality theory promotes the investigation of ‘intra-actions’ and assemblage dynamics in hominin object environments as well as the conactive coupling of humans and materials, NCT primarily focusses on material inertia and ecological feedback loops (Tomlinson 2018; Riede 2019). Yet, both perspectives recognise the historical efficacy of changing material worlds and the need to examine them as evolutionary *input variables* with important long-term effects.

While not to be conceptually equated, NCT and object-centred approaches may thus profitably join forces and inspire yet also challenge each other in the study of long-term material culture change. Both illustrate the capacity of past hominins—alongside many other organisms—to construct, modify and select biotic and abiotic components of their local environment (Laland and Sterelny 2006; Laland *et al.* 2008) and to engage in ‘ecosystem engineering’ (Jones *et al.* 1994), while in part surrendering control, foresight and authority over the ensuing trajectories. The agency of objects, object assemblages and object-worlds therefore flags up a promising arena of dialogue and exchange between ‘thick’ analysis in the humanities tradition and ‘science-based’ research in evolutionary archaeology (*cf.* Riel-Salvatore 2010; Riede 2011, 2019). To paraphrase Bennett (2004), the materiality of hominin-inherited object environments gives birth to a matrix of ‘material recalcitrances’ which powers, and at times impels, the millennial-scale evolution of hominin biocultural and technical productions and orientates archaeological research towards the *self-referential* (Luhman 1990) and *autocatalytic* properties of evolving material worlds (Simondon 1958; Deacon 2011). At least four consequences of long-term material pre-furnishment in human evolution should be examined here: (1) The ability of pre-allotted object-scapes and thing-worlds to catalyse, and potentially determine, the problem–solution and innovation behaviour of their hominin inheritors; (2) the capacity of material worlds to reinforce, fixate and conjugate trajectories of object maintenance and optimisation; and (3) the proclivity of pre-invested material environments to configure and structure their own development, *e.g.* by introducing new needs, facilitating discoveries and determining which changes and novelties may favourably be integrated into existing object assemblages (and which are not); and (4) the role of thing-worlds in influencing and altering human–environment dynamics.

Object-oriented research may help to overcome ahistorical conceptualisations of hominin problem-solving as propagated by the metaphor of the engineer, which re-casts problem-solving as a question of independent design: The problem-solver, then, merely has to optimise the matching relationship between a given problem and the offered solution (*cf.* Hussain 2018a, p. 219). Through the lens of the engineer, problem-solving is all about finding the *ideal solution* and the latter should in principle not depend on input variables. It is the *unconditional* performance of the offered solutions which emerges as the prime measure of innovative success. Taking thing-powers and material vibrancies into consideration supplies an alternative problem–solution metaphor: The *bricoleur* or *tinkerer* (*sensu* Lévi-Strauss 1966, p. 19; *cf.* Johnson 2012). Instead of chasing ideal solutions, the bricoleur operates with the resources afforded by her/his material world and actively explores their developmental and combinatory possibilities (Duymedijan and Rüling 2010; Sanchez-Burks *et al.* 2015). Bricolage is an eclectic, diffuse and inherently praxis-oriented concept, which recognises the *creative potential* of heterogeneous physical and biocultural environments (*cf.* Webster 2019). Problem–solution behaviour is viewed here as the experimental and situational mobilisation, re-combination and refinement of pre-existing resources to make something work and to discover new tenable ways forward. This perspective helps in recognising the materiality of object environments as a basic *scaffolding mechanism* (Wood *et al.* 1976, p. 98) for problem-solving, innovation and learning in human evolution (Orlikowski 2006). Material scaffolds may be defined here as key ‘ecological and adaptive supports channelling and facilitating problem-solution behaviours through artefacts, technologies and material infrastructures’ (*cf.* Strömmer 2016, p. 242–244). The material world, from this point of view, is much more than an inert backdrop or a pre-condition of human solution-finding and innovation. Distinct object assemblages and confederacies of materials, technologies and hominins can give birth to original patterns of discovery, innovation and adaptive refinement, and an archaeology of innovation remains incomplete if the materiality of the contributing things and objects is not explicitly theorised and examined.

Understanding problem–solution behaviours and material culture innovation through the prism of the bricoleur has important ramifications for the rationality and optimality theorem guiding many flagship approaches in evolutionary and ecological archaeology (*e.g.* Bleed 1986; Torrence 1989; Bamforth and Bleed 1997) and may help to overcome some of their limitations (*cf.* esp. Jochim 1998, p. 223): The conactive involvement of materials and objects in the discovery, design and implementation of new concepts and technologies easily fosters ‘sub-optimal’ or ‘less-efficient’ solutions and spawns messy patterns of long-term coping. Materiality theory assists in the explanation of these patterns and shifts the attention from processes of ‘external optimisation’—the close-to-perfect match between problem and engineered solution—to ‘internal optimisation’ centred on pre-invested material worlds and their derived historical potential for re-combination, exaptation and cross- and self-adaptation as well as playful experimentation (Garud *et al.* 2010, 2016; Nowell 2016; Riede *et al.* 2018). The guiding rationality model is not ‘objective’, ‘global’ and ‘absolute’ but instead ‘situated’, ‘local’ and ‘bounded’ (*cf.* Simon 1997; Gigerenzer and Selten 2001), and is anchored in changing object heuristics and the material fabric of evolving hominin lifeworlds. While traditional evolutionary approaches routinely focus on the adaptive roots of hominin problem-solving, object-oriented perspectives point to the fact that many realised solutions of the past may be ‘quick fixes’ and—albeit perhaps less-optimal—have the ability initiate path dependencies with difficult-to-escape long-term socio-material consequences.

Notwithstanding its originality, the bricolage model of problem-solving exhibits some notable convergences with newer developments in evolutionary thinking, highlighting path dependencies and developmental constraints (*e.g.* Gould and Lewontin 1979; Gould 2002; Szathmáry 2006) or advocating the image of rugged, multi-dimensional fitness landscapes (Wright 1931; Pigliucci 2008; Will *et al.* 2019). In the latter view, organisms regularly fail to find the optimal peaks of a given fitness landscape—*e.g.* due to pervasive constraints of their phylogenetic, ecological and material inheritance as well as of their adaptive material environments—and more often than not end up inhabiting sub-optimal locations. This perspective on long-term material culture change is supported by many classic studies on the history and evolution of human technology (*cf. e.g.* Basalla 1988; Rogers 1995; Arthur 2009): Long-term trajectories of technological change are often characterised by largely continuous improvement and synthesis upon already existing objects, ideas and solutions, rather than revolutionary leaps forward made possible by some genius engineering inventors.

Object-centred approaches to long-term patterns of the human deep past also defuse simple models of correlation and environmental determinism, which enjoy continued popularity among Palaeolithic archaeologists (Livingstone 2012), and opens up new avenues for interrogating the human–environment nexus. Materiality theory undermines the long-standing assumption that the environmental context of human evolution constitutes an unconditional first principle (*cf.* Potts 1998, 2012; Finlayson 2004, 2009; Petraglia *et al.* 2009) and calls attention to the explanatory significance of *material mediation* and *translation*: Given the growing dependency of hominins on their technological worlds in the course of human evolution (Shea 2016, 2017) and the inescapability of pre-allotted object-scapes, environmental adaptation naturally hinges on the nature, structure and potentiality of these object ecologies. The role of natural environments depends on pre-furnished options and material affordances provided by material environments. Not only do socio-material starting conditions—often historically contingent—greatly matter then (*cf.* Valentin 2008; Audouze and Valentin 2010; Marchand 2014), possible and ideal technological adaptations will not always prove feasible, implementable or even discoverable under the given material conditions. Object-oriented perspectives hence subvert unidirectional models of human–environment relations and place the often fragile, imperfect and tension-ridden *coordination* of material possibilities and environmental constraints at the centre of analysis: Technological adaptation comes into view as a long-term *effort* or *process* rather than an event or condition, and both climate and environment are relegated to a ‘catalystic’ role (*sensu* Morsink 2019) in lithic evolution (see Bon 2009, p. 243 for a similar critique). Instead of falling back into rigid epistemological camps (environmental determinism, possibilism and relativism), this perspective paves the way for a comparative and scenario-driven re-evaluation of the relative contribution of environmental, material and hominin agencies in long-term palaeoarchaeological pattern formation.

Hopkinson’s (2004) account of the emergence of Late Middle Palaeolithic (LMP) leafpoint assemblages offers an opportunity to probe into this promise of object-oriented research in lithic studies. Hopkinson (2004, 248) argues that the appearance of leafpoint assemblages at the end of the European Middle Palaeolithic is linked to shifting rates of environmental change, especially pulses of forestation and deforestation, which triggered specific responses in hominin mobility and technology. He (*idem*) proposes that leafpoints fulfilled task-specific needs and thus embody a direct adaptation to increasingly fragmented landscapes, in which hominin land-use became progressively radiated. Even though Hopkinson’s account specifies the functional relationship between climate, landscape, mobility and lithic technology, it falls short in explaining the *historical specificity* of the adopted leafpoint solution due to a neglect of the processual dynamics and material pre-conditions of their supposedly adaptive design. In other words, it remains unclear why other possible technical and behavioural solutions should be considered less favourable given the environmental circumstances. Two observations are important here: First, leafpoints appear in notable numbers only at the distal end of the LMP cycle and join a broader lithic environment in which various other bifacial tools are foundational infrastructural elements (Richter 2009; Kot and Richter 2012). Second, small numbers of leafpoints or leafpoint-like objects are already encountered in LMP contexts preceding the leafpoint-rich layers in question (*cf.* Bosinski 1967; Richter 2018, p. 174). From a materiality perspective, this suggests that the idea of the leafpoint was already implicit in the broader design space of LMP bifacial production and that the potential of discovering the benefits of the leafpoint and formalising its design was elevated in a material world anchored in diversified bifacial technologies.

In this view, the LMP leafpoint neither represents a *de novo* invention, nor does it substantially deviate from the techno-logic, design and instrumentalisation modalities of other co-existing and already-in-use object groups of its larger temporal horizon. The total matrix of LMP object-scapes and technical knowledge, in other words, was pre-configured in such a way that the concept of the leafpoint could easily be discovered, exapted and refined without the need to develop a whole new suite of technical and cognitive resources or supporting material infrastructures. In fact, LMP object-scapes would have actively encouraged the implementation of leafpoint technology and provided plenty of synergistic opportunities for it to flourish. The formalisation of the leafpoint concept further resonates with the general tendency of increasing structural differentiation of bifacial assemblages in the LMP. Instead of reflecting an optimal or engineered solution to cope with increasing landscape fragmentation and climate variability at the end of MIS 3, the leafpoint phenomenon is thus perhaps more parsimoniously understood as the *coordination* of the inherent material potential of pre-leafpoint LMP worlds, their tendency of ‘structural deepening’ (*sensu* Arthur 2009; see above) and external environmental changes. This alternative account also helps to make sense of the discontinuous spatiotemporal pattern of leafpoint-bearing assemblages at the end of the LMP without resorting to Hopkinson’s (2004) stranded assertion that the knowledge of making these technical objects must have been memorised over millennia.

Materiality concepts may also help to resolve a range of taxonomic, chronological and interpretive issues, balancing, and if necessary countering, persistent anthropocentric or ecocentric tropes in the field. For example, the controversy surrounding the so-called M.M.O. (*Mousterian with Micoquian Option*; *cf.* Jöris 2004, p. 90) may become superfluous as soon as the M.M.O is framed, in a similar fashion as other archaeological macro-phenomena discussed before, as a long-term *material disposition* with an inherent yet not always realised potential for object specialisation, miniaturisation and bifacial refinement. This reading of the M.M.O. is compatible with the original idea motivating its formulation (Richter 1997, 2018, p. 132; Uthmeier 2004): The tendency of Mousterian technological worlds to author emergent patterns of lithic shape modification and transformation, often supplying distinct assemblages of bifacial tools and Upper Palaeolithic forms whose appearance in a given assemblage depends on a host of contextual factors, such as raw material supply, task differentiation and possibly the history and intensity of hominin occupation. Fuzzy, unstable and transformative lithic patterns are in this way decoupled from universal hominin behavioural or cognitive schemata, and techno-typological specificities can be re-cast as technical extremities, nodal points or distal ends on a spectrum of options provided by the hominin-inhabited material worlds we designate ‘Mousterian’. An object-oriented perspective helps to embrace the M.M.O. as a processual *negotiation* of thing-powers, assemblage dynamics and evolving hominin horizons rather than as a static culture-historical entity (as *e.g.* in Ruebens 2013).

Along broadly similar lines, materiality theory may be deployed to re-invigorate some of the long-standing origins questions in the history of (lithic) technology. These include the hypothesised evolutionary relationship between LCTs and the first proper handaxes of the Lower Palaeolithic (Bordes 1971; Beyene *et al.* 2013), the link between bifacial reduction and the emergence of prepared core technology (Tryon 2003; Tryon *et al.* 2005; Villa 2009; Adler *et al.* 2014) or the proclaimed evolutionary bond between ‘Proto-Levallois’ technology of the Victoria West type and the emergence of full-blown Levallois reduction architectures (Van Riet Lowe 1929; Breuil 1930; Kuman 2001; Sharon and Beaumont 2006). Object-oriented research helps to elucidate similarities and differences in the material logic of the respective technological solutions and helps to critically and creatively reflect on the *material conditions* under which new technologies may have been discovered, implemented and developed further. In doing so, it pays close attention to the possibilities and affordances of different reduction principles and the opening up or closure of design spaces (*cf.* Moore 2011; McGhee 2018). The genesis of key technologies—currently a puzzling theme in Palaeolithic scholarship—may then be placed on more solid theoretical grounds and re-evaluated as a process structured and pre-configured by inherited and evolving material ecologies.

## Conclusion and Perspectives

The aim of this paper was to explore the potential of object-centred approaches and concepts borrowed from materiality theory to inform the study of the human deep past. We have shown that in contrast to widespread belief among Palaeolithic archaeologists, object-oriented explorations provide hands-on, non-esoteric and widely applicable perspectives on early human prehistory and supply a wealth of new questions and hypotheses on the evolution of our species. At the core of the materiality project is an attempt to de-centring the human, environmental and cultural in order to analyse the genuine contribution of material things, artefacts and technologies. For Palaeolithic archaeology, the key realisation is that hominins and things have continually made and re-made each other over many thousands and millions of years. The human condition is thus as much a product of thing-powers as of our own derived capacities, behaviours and achievements. Materiality approaches promote an integrated and pluralistic understanding of the Palaeolithic past as a *co-production* of humans and non-humans and demonstrate their complex and deep-historical entanglement, rooted in ever-shifting power relations and mutual influences. Materiality theory underscores that even human-made objects and technologies regularly escape the realm of human control, and the unique observational scales of the Palaeolithic—global in scope and tempered by millennia—provide privileged access to these material claims with often large-scale repercussions for the course of human history.

**Fig. 8:**
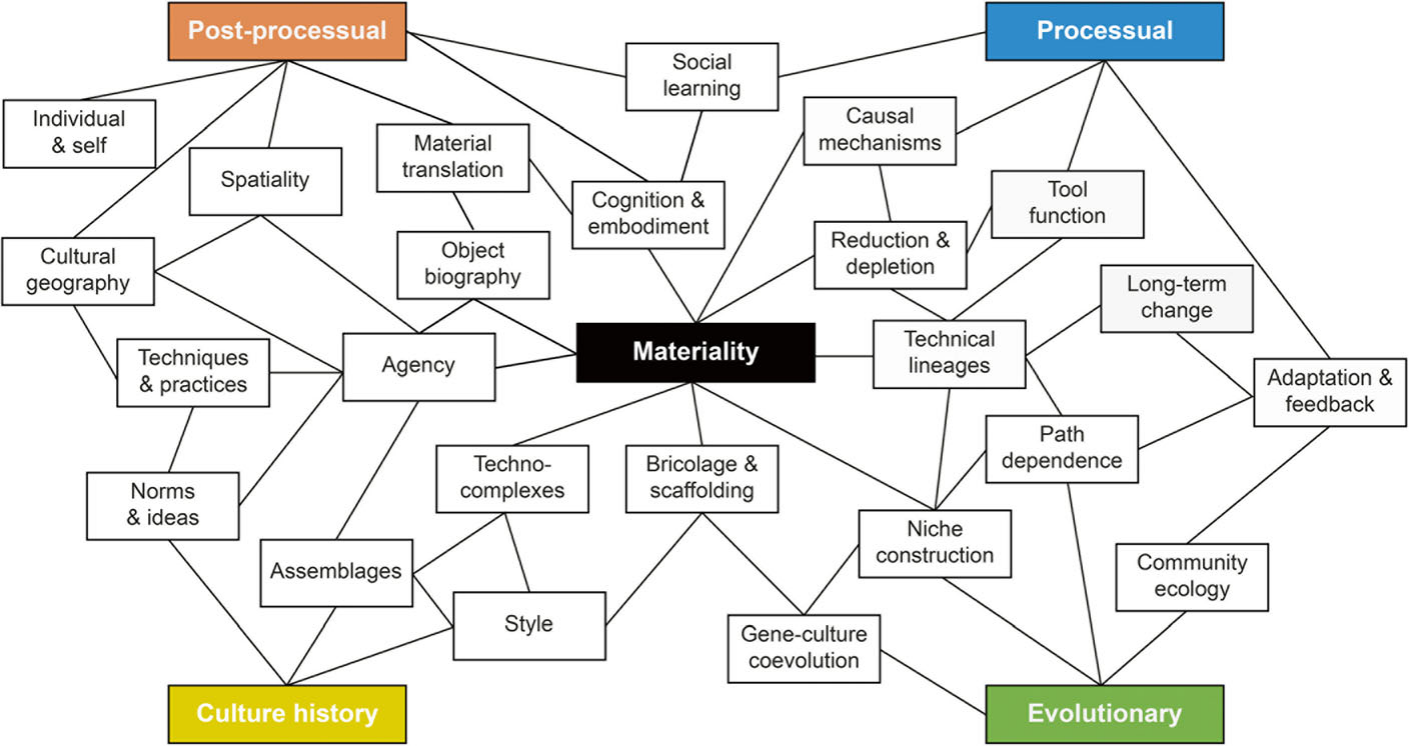
Network diagram showing the relationship between materiality theory and traditional areas of inquiry in Palaeolithic archaeology, organised according to broader culture-historical, processual, post-processual and evolutionary research orientations

The reflections, explorations and case studies on the manifold roles, impacts and consequences of material things throughout the Palaeolithic offered in this paper are not intended as a comprehensive or conclusive inventory, but are instead provided in the spirit of introduction to map out the terrain of fruitful engagement with the materiality of the deep past. The goal was to sketch the interpretative possibilities and to supply readers with concrete examples to evaluate whether or not materiality theory can make a valuable contribution to Palaeolithic archaeology and human origin studies and how it may be used. We hope to have illustrated that object-oriented perspectives can not only complement and refine existing knowledge and fill some of its gaps but also offer an original inroad to the formative dynamics that underpin the observed patterns of the record, providing a useful means of critiquing and disrupting received interpretations and understandings in the field. The here sketched materiality project neither supports partisanship nor exclusionary politics of science. Instead, our venture provides a dynamic and multi-stranded platform for intra- and cross-disciplinary dialogue and inspiration (Fig. 8). Materiality theory tables new incentives to broaden and reconsider the scope of traditional Palaeolithic research by incorporating the study of the role of objects, assemblages and object-worlds in the making of the hominin past and by motivating new disciplinary alliances and forms of cooperation. As such, scholars do not need to subscribe to all of the claims and arguments made in this paper. The aspiration was rather to promote a new object-oriented discourse in the field and to draw attention to the many insights that scholars of materiality can glean from scrutinising the Palaeolithic archaeological record.

As an open-ended and multi-paradigmatic enterprise, materiality yields the promise of synthesising a large array of already existing information, to bridge polarised debates and to motivate new ways of thinking about the Palaeolithic past. At the very least, materiality theory can assist Palaeolithic archaeologists in finding more productive questions, disclosing hidden biases and sharpening their arguments and hypotheses. The fresh perspectives on macro-archaeological signatures and putative taxonomic units as well as human–environment dynamics discussed in this paper illustrate this potential. These examples also demonstrate that object-centred approaches productively re-orientate research to the material conditions of the distant past and force scholars to consider and re-imagine the consequences of hominin life and evolution in structured material environments.

The materiality nexus cross-cuts theoretical schools and research traditions and reveals otherwise easily overlooked meta-theoretical convergences between counteracting positions in the field. It therefore offers a welcome opportunity to re-ignite the conversation between humanities-based and science-based approaches, *e.g.* in evolutionary archaeology where the idea of evolving technological lineages or object assemblages with consequential histories for human populations is now slowly gathering momentum (*e.g.* Shott 2011; Shennan 2013). Some branches of community ecology and especially NCT similarly yield dynamic and history-infused readings of the material past, emphasising the active role of objects and material environments in human evolution (Laland and O’Brien 2010; Riede 2019), and gravitate towards some of the long-standing ideas and theories from the forefront of object-oriented scholarship in the humanities (*cf.* Boivin 2008, p. 181–224; Tomlinson 2018). All of these approaches and perspectives share a concern with the possibilities and consequences of living in and adapting to changing object ecologies, and they all recognise the difference-making capacity of material surroundings: Stone artefacts and larger artefact-worlds *evolve* and have a *history of their own* and this history greatly matters for the behaviour and evolution of the appendant hominins.

Yet even more, Palaeolithic archaeology stands to make an original contribution to the transdisciplinary development and expansion of materiality theory (Bennett 2010; DeLanda 2016) as well as multispecies and more-than-human studies (Haraway 2007; Grusin 2015). The Palaeolithic record provides countless possibilities to investigate thing-powers and human–thing relations on temporalities and geographical scales inaccessible to other human sciences. The human deep past further promotes studies of the co-evolutionary interplay and trade-off dynamics between biological, environmental and material factors (*e.g.* Key *et al.* 2020), refining our grasp on the role of object ecologies and material tendencies in the making of different surviving and fossil humanities (*e.g.* Neanderthals, Denisovans, *etc*.). This deep-time and multispecies perspective on object agencies considerably extends the scope of materiality discourses in the humanities. It also provides an impetus to broaden the scope of the EES even further to supply some additional building blocks for the development of a general theory of evolution (*cf.* Schurz 2011), in which distinct field forces (material, social, biological, cognitive) are recognised in the same breath as multi-level selection, synergy and lateral gene transfer (*cf.* Corning 2005).

The advancement and proliferation of life-scientific and geochemical fingerprinting techniques, such as ZooMS and XRF, as well as non-invasive screening methods such as Raman spectroscopy, offer another opportunity to transform the investigation of the changing material conditions of human evolution. Together with approaches and perspectives borrowed from materials science, these methods tender new means of examining the intersection between material configurations, object qualities and design and human socio-cultural behaviour. They help to tackle long-standing questions about Palaeolithic human–material–animal conactivities and their socio-cultural significance (*cf. e.g.* McGrath *et al.* 2019; Martisius *et al.* 2020). The integration of object-oriented arguments and materiality reflections therefore amplifies current lines of investigation, helping them to exploit their full analytical and interpretive potential and stimulating novel readings as well as previously unrecognised cross-disciplinary synergies. Because of the traditionally important role of bio- and life-scientific approaches in wider human origin studies, Palaeolithic archaeology might be regarded as a crucial testbed for applying these methods and techniques and bring them into dialogue with interpretive concepts drawn from materiality thinking in the humanities and social sciences.

Materiality theory is not simply interpretive or post-processual archaeology in disguise or relevant only for the study of hominin cognition, symbolism and art-making. Object-oriented reasoning and analysis rather emerge as an original window into almost all dimensions of the Palaeolithic past, including such seemingly mundane practices as the manufacture, utilisation, transformation and discard of stone tools and implements. Object-oriented research thereby not necessarily implies a radical break with established and long-standing approaches, interpretations or viewpoints. Instead, it often helps to consolidate previous observations and insights, to support them with novel concepts and theories and to work towards a new synthesis of findings. Nonetheless, the shift in perspective induced by materiality thinking can at times be far-reaching and—as we have tried to show here—often inverts long-standing interpretations and narratives of the human deep past. Given the astonishing speed of knowledge expansion and methodological innovation in Palaeolithic archaeology, it seems timely to seize the opportunities offered by the growing body of thing theories and to integrate them into ongoing Palaeolithic research. It is time to recognise the materiality, agency and evolution of objects, assemblages and technologies as a self-sufficient area of inquiry in the field.
